# Prelacteal feeding and its relationship with exclusive breastfeeding and formula consumption among infants in low- and middle-income countries

**DOI:** 10.7189/jogh.12.04104

**Published:** 2022-12-23

**Authors:** Paulo AR Neves, Nancy Armenta-Paulino, Luisa Arroyave, Luiza IC Ricardo, Juliana S Vaz, Cristiano S Boccolini, Linda Richter, Rafael Peréz-Escamilla, Aluísio JD Barros

**Affiliations:** 1 Centre for Global Child Health, The Hospital for Sick Children, Toronto, Canada; 2 International Center for Equity in Health, Universidade Federal de Pelotas, Pelotas, Brazil; 3 Center for Health Systems Research, National Institute of Public Health, Cuernavaca, Morelos, Mexico; 4 Surgery and Medical and Social Sciences Department, University of Alcalá de Henares, Alcalá de Henares (Madrid), Spain; 5 Faculty of Nutrition, Universidade Federal de Pelotas, Pelotas, Brazil; 6 Institute of Scientific and Technological Communication and Information in Health, Fundação Oswaldo Cruz, Rio de Janeiro, Brazil; 7 DSI-NRF Centre of Excellence in Child Development, University of the Witwatersrand, Johannesburg, South Africa; 8 Yale School of Public Health, Yale University, New Haven, Connecticut, USA

## Abstract

**Background:**

Early feeding practices are important determinants of optimal feeding patterns later in life. We aimed to investigate if giving any fluids or foods other than breast milk during the first three days after birth (prelacteal feeds) affects exclusive breastfeeding and consumption of formula among children under six months of age in low and middle-income countries (LMICs).

**Methods:**

We conducted a retrospective cohort study using data from 85 nationally representative Demographic Health Surveys (DHS) and Multiple Indicator Cluster Surveys (MICS) in LMICs (2010-2019). We considered three exposures: any prelacteal feeding (PLF), milk-based only prelacteal feeding (MLK), and water-based only prelacteal feeding (WTR), according to the DHS/MICS definition. The outcomes were exclusive breastfeeding, based on the World Health Organization definition, and consumption of formula among infants under six months of age. We used Poisson models adjusting for sociodemographic indicators, antenatal care, birth assistance, and early initiation of breastfeeding to estimate the effects of the exposures on the outcomes. Findings were grouped by each country, as well as by regions of the world and national income classification.

**Results:**

We included data from 91 282 children. PLF, MLK, and WTR had a prevalence of 33.9% (95% confidence interval (CI) = 33.6-34.2), 22.2% (95% CI = 21.9-22.4), and 9.4% (95% CI = 9.2-9.6), respectively. Exclusive breastfeeding and consumption of formula had a prevalence of 35.2% (95% CI = 34.9-35.5) and 27.7% (95% CI = 27.4-28.0), respectively. In the crude analysis, children who were given PLF were 40% less likely to be exclusively breastfed (prevalence ratio (PR) = 0.60; 95% CI = 0.56-0.64) and nearly twice more likely to receive formula (PR = 1.89; 95% CI = 1.72-2.08); the direction of the associations was the same across income groups and regions of the world. In the adjusted analysis, the observed crude effects were only slightly reduced (exclusive breastfeeding – PR = 0.62; 95% CI = 0.59-0.66, consumption of formula – PR = 1.72; 95% CI = 1.59-1.85). MLK showed a stronger impact on the outcomes than PLF, especially for formula consumption (adjusted PR = 1.81; 95% CI = 1.67-1.97) and in low-income countries. WTR was only negatively associated with exclusive breastfeeding (adjusted PR = 0.69; 95% CI = 0.63-0.75), but not with formula consumption (adjusted PR = 1.09; 95% CI = 0.99-1.20).

**Conclusions:**

Feeding babies prelacteal foods shortens exclusive breastfeeding duration and increases the likelihood of formula consumption in children under six months of age in LMICs. Pro-breastfeeding interventions must be prioritized during antenatal care and throughout the stay in the maternity facility to properly protect, support, and promote exclusive breastfeeding since birth.

The World Health Organization (WHO) defines prelacteal feeding as any fluid given to a child before breastfeeding starts [1]. However, a standard definition is lacking, while a few guidelines also consider prelacteal feeds as the introduction of foods before copious amounts of breast milk start to be produced [2]. Despite such differences, prelacteal feeding is the introduction of foods and/or liquids other than breast milk in the first few days after birth [3].

Prelacteal feeding is a frequent practice in both high-income and low- and middle-income countries (LMICs) [4-7]. In an analysis considering 72 LMICs, United Nations Children’s Fund (UNICEF) estimated that 25.0% of newborns received water-based and 18.0% milk-based prelacteal feeds between 2010 and 2014 [4]. Recent analyses using data from nationally representative surveys in LMICs estimated that 30.0 to 50.0% of children under two years of age received prelacteal feedings in the first three days of life [5,6]. Prelacteal feeding is detrimental to optimal breastfeeding practices. In a meta-analysis of 27 longitudinal studies from high and low resource settings, prelacteal feeding was significantly associated with lower exclusive breastfeeding (relative risk = 1.44, 95% confidence interval (CI) = 1.29-1.60) and any breastfeeding cessation rates (relative risk = 2.23, 95% CI = 1.63-3.06) among infants younger than six months of age [7].

The infant formula is one type of prelacteal feeding normally used in hospital settings [5-7]. We are unaware of any previous study that used global pooled national-representative data to investigate if the likelihood of a child receiving infant formula feeds later in life increased when the child was exposed to prelacteal feeds. This investigation is important considering the aggressive and ubiquitous commercial milk formula (CMF) marketing worldwide [8,9]. Furthermore, in the last two decades, CMF consumption has increased in LMICs, but especially in upper-middle income countries [10].

Optimal breastfeeding practices improve the health and well-being of children, women, and society as a whole [4,11,12]. The WHO recommends children to be put to the breast within the first hour after birth, to be exclusively breastfed during the first six months of age, and to continue to be breastfed for at least two years while they receive adequate and healthy complementary feeding [12]. Despite the public health relevance of breastfeeding, less than 50.0% of newborns are put to the breast in the first hour after birth in LMICs [4,5] and only 48.6% of infants under six months of age were exclusively breastfed in 2019 [10]. Although prelacteal feeds have been associated with suboptimal breastfeeding practices, an analysis aimed to investigate this association based on a large number of LMICs, to our knowledge has not been previously undertaken. Moreover, the impact of prelacteal feedings exposure on the consumption of CMF has also not been investigated following this approach. For this reason, we aimed to investigate if giving prelacteal feeds (ie, fluids of foods offered during the first three days after birth) affects exclusive breastfeeding practices and consumption of CMF among children under six months of age in LMICs. We also sought to explore if the association between prelacteal feeding and infant and young child feeding indicators differs by type of prelacteal, ie, milk-based or water-based.

## METHODS

This is a retrospective cohort study based on nationally representative surveys carried out in LMICs that contain information on both infant and young child feeding practices and prelacteal feedings – the Demographic Health Surveys (DHS) [13] and Multiple Indicator Cluster Surveys (MICS) [14]. Such surveys collect data on a vast set of reproductive health, maternal, newborn, and child health and nutrition indicators, employing multi-stage sampling procedures to collect data at household level. We also used information from the nationally representative surveys carried out in Peru (Encuesta Demográfica y de Salud Familiar 2019) [15] and Bolivia (Encuesta Demográfica y de Salud 2016) [16] after harmonization of the data sets according to DHS/MICS standards [17]. Trained field workers applied standardized questionnaires through face-to-face interviews with women of childbearing age (15-49 years) regarding child dietary practices in the previous day for the youngest child born in the two years preceding the survey [18]. Following WHO and DHS recommendations, missing values and “don’t know” answers to the feeding indicators were considered as not consumed [19,20]. The percentage of missing information was 5.0% for prelacteal feedings, 3.7% for exclusive breastfeeding, and 2.8% for formula consumption.

This analysis included the most recent survey for each country carried out in 2010 or later that contains information on the variables of interest. Figure S1 in the **Online Supplementary Document** details the flow-chart illustrating the selection of surveys.

### Exposures and outcomes

The main exposure was any prelacteal feeding defined according to the DHS/MICS convention, measured with the following question: was the child ever breastfed and given any type of liquid, but not breast milk (formula, other animal milk, water, tea, juice, soup etc.) in the first three days after birth [5,20-22]? Although we acknowledge that this definition does not capture the previous one from the WHO [1], this is the most pragmatic way to identify prelacteal feedings in such large national surveys [5]. In DHS/MICS surveys, any prelacteal feeding is a construct built based on information about two different types of prelacteals. Therefore, to distinguish the effects of different types of prelacteals on the outcomes, we also investigated the following secondary exposures: milk-based only prelacteal feedings (was the child ever breastfed and given only milk-based liquids (formula and animal milk, not considering breast milk) in the first three days of life) and water-based only prelacteal feedings (was the child ever breastfed and given water-based only liquids (water, tea, honey, juice, sugar water, etc.) in the first three days of life).

The outcomes of interest were exclusive breastfeeding and consumption of CMF among children under six months of age (0-5 months). We used the WHO definition for exclusive breastfeeding [23]: the child was fed exclusively with breast milk during the day before the survey, except for prescribed medicines and micronutrient supplements, and not including no other food or drink, not even water. CMF consumption was defined as the child receiving a formula milk feed the day before the interview [10].

### Covariates

The following additional variables were adjusted for in the models: household wealth (quintiles), area of residence (urban or rural), mother’s level of education, mother’s age, number of antenatal care visits (less or more than four visits), skilled birth attendant (yes or no), institutional birth (home or institutional birth), caesarean section birth (yes or no), sex of the child (girl or boy), and early initiation of breastfeeding (yes or no). The wealth index was already available in the surveys’ data sets and was calculated based on the presence of household assets (car, television, radio, etc.) and home infrastructure (presence of toilet, electricity, building characteristics, etc.) [24,25]. Separate principal component analyses were carried out for urban and rural households and then later combined into a single score using a scaling procedure that allows comparability between the areas of residence [26]. The final wealth index was split into quintiles, where the first quintile represents the poorest households and the fifth quintile the richest households. The definitions of area of residence and mother’s formal education are country-specific and provided in the raw data sets. We recoded the level of education of mothers into three categories: none (no formal education), primary (seven years or less), and secondary (eight years or more). We grouped the mother’s age at the time the survey as between 15-17 years, 18-19 years, and 20 years or more. We defined the early initiation of breastfeeding as the child being put to breast within the first hour after birth [18].

### Statistical analysis

We calculated the national prevalence of the feeding indicators for each survey included and by each exposure deemed in the analysis. Negative 95% CI values were truncated at zero. We developed a directed acyclic graph to guide the analysis of the relationship between the variables under study using the DAGitty v3.0 software [27] (Figure S2 in the **Online Supplementary Document**).

Poisson regression has been frequently used with binary outcomes in place of logistic regression since the early 2000s to directly estimate prevalence ratios (PRs ) which are more interpretable than odds ratios. This is especially true with common outcomes [28]. We thus used crude and adjusted Poisson regression models with robust variance to estimate PRs and 95% CIs for the associations between types of prelacteal feeds and analysed outcomes. For selection of covariates in the adjusted models, we performed a stepwise approach retaining covariates associated with the outcomes presenting a *P*-value of <0.10. All study covariates were significantly associated with the outcomes at this level.

We investigated collinearity through Pearson correlation and the post-estimation variance inflation factor and only found evidence of a lack of independence between skilled birth attendant and institutional birth. Hence, we opted to drop skilled birth attendant out of the models due to the direct relationship between institutional birth and prelacteal feeding [5]. For children not exclusively breastfed, we calculated the proportion of different food types consumed in the day before the survey by exposure to milk-based and water-based only prelacteals. The following food groups were considered: any breastfeeding, plain water, other liquids (sugar water, juices, liquid soups/clear broth, and other liquids), other milks (formula or animal milks (cow, goat, etc.)), and complementary foods (baby food, flesh, eggs, vegetables, fruits, yogurt and dairy, tubers and grains, and other solid-semisolid foods).

We grouped the analysis by world regions using the UNICEF regional classification and the World Bank income groups classification corresponding to the median year the surveys were carried out [29]. The effect of the exposures on the outcomes by world regions and income groups was assessed by pooling PR estimates for each country within each UNICEF region or income group using a meta-analysis approach with random effects, with weights reflecting survey sampling size. The *I*^2^ statistics is presented as a measure of heterogeneity. We used Stata 17.0 (StataCorp, College Station, TX, USA) to perform the analysis based on individual-level data and considered the complex design of the surveys using the svy command.

### Ethics

Our analysis is based on anonymous information and publicly available data sets. The institutions conducting the original surveys were responsible for the ethical clearance.

## RESULTS

Among the 99 surveys with data available in the database maintained by the International Center for Equity in Health, we deemed 94 as eligible. We had to exclude nine surveys due to imprecise estimates obtained after running the models, mostly because convergence was not achieved (too few children with concomitant exposure and outcome data). For the main exposure analysis, we included 85 surveys (median year = 2015; Table S1 and Figures S1 and S3 in the **Online Supplementary Document**). However, for the secondary exposures, this number dropped due to the lack of convergence of the models; analyses of milk-based only prelacteals were based on 79 surveys and those of water-based only prelacteal feeds on 71 surveys (Figure S1 in the Online **Supplementary Document**).

We included a total of 91 282 children under six months of age in our sample. The children were mostly from rural areas (66.8%). They belonged to the two poorest quintile groups (48.7%) and approximately half were male (50.9%). Almost half of children were put to the breast within the first hour after birth (49.3%). 88.6% of mothers were ≥20 years old, 46.5% completed secondary educational level, 55.6% attended four or more antenatal care visits, 85.5% did not undergo a caesarean section birth, and 72.0% delivered the children in a health institution (data not shown in tables).

The pooled prevalence of the exposures and outcomes for all LMICs in this analysis were, as follows: any prelacteal feed – 33.9% (95% CI = 33.6-34.2); milk-based only prelacteal feeds – 22.2% (95% CI = 21.9-22.4); water-based only prelacteal feed – 9.4% (95% CI = 9.2-9.6); exclusive breastfeeding – 35.2% (95% CI = 34.9-35.5); and CMF consumption – 27.7% (95% CI = 27.4-28.0). For the exposures, the countries with the highest and lowest prevalence for any prelacteal feeding were Chad (86.8%) and Zambia (5.0%); North Macedonia (57.1%) and Eswatini (0.0%) for milk-based only prelacteal feeding, and Chad (71.3%) and Montenegro (0.0%) for water-based only prelacteal feeding. For the outcomes, the countries with the highest and lowest prevalence of exclusive breastfeeding were Zambia (72.5%) and Chad (0.3%); for CMF consumption, they were Gabon (63.5%) and Burkina Faso (0.8%) (Table S2 in the **Online Supplementary Document**). Table S3 in the **Online Supplementary Document** shows the percentage of children exclusively breastfed and the percentage who consumed CMF under six months by prelacteal feeding exposures.

**Figure 1:**
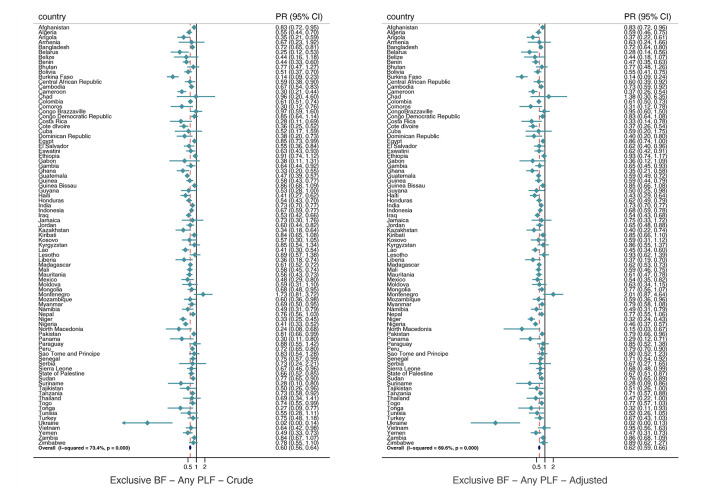
Pooled and country-specific prevalence ratios of the effect of any prelacteal feeding on exclusive breastfeeding. Exclusive BF – exclusive breastfeeding, any PLF – any prelacteal feeding.

**Figure 2:**
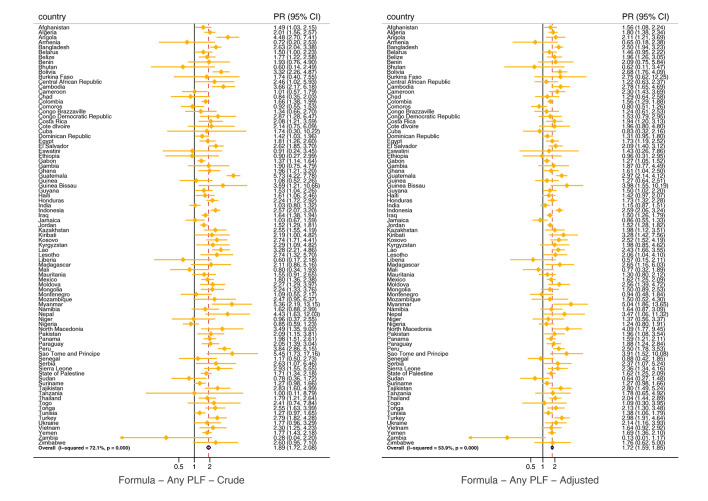
Pooled and country-specific prevalence ratios of the effect of any prelacteal feeding on formula consumption. Formula – formula consumption, any PLF – any prelacteal feeding.

In the crude analysis, any prelacteal feeding was inversely associated with exclusive breastfeeding in the pooled analysis (PR = 0.60; 95% CI = 0.56-0.64) and in all world regions and national income groups. The findings changed slightly when the model was adjusted for covariates in the pooled (PR = 0.62; 95% CI = 0.59-0.66) and sub-groups analysis (**Figure 1**; Table S4 in the **Online Supplementary Document**). For CMF consumption, we observed a direct and significant crude association with any prelacteal feeding when pooling all countries (PR = 1.89; 95% CI = 1.72-2.08) and when conducting the analysis by world regions and countries’ income groups. After adjusting for covariates, the results changed marginally in the all-countries joint estimate (PR = 1.72; 95% CI = 1.59-1.85) and in the analyses by income groups and world regions, except for Eastern and Southern Africa where the estimates were no longer significant after adjustment (**Figure 2**; Table S4 in the **Online Supplementary Document**).

For the secondary exposures, milk-based only prelacteal feedings were inversely and significantly associated with lower rates of exclusive breastfeeding in the pooled analysis (crude – PR = 0.69; 95% CI = 0.64-0.73; adjusted – PR = 0.73; 95% CI = 0.68-0.78) and in the analyses across all income groups and world regions, except for West and Central Africa. The association between milk-based only prelacteal feeding and CMF consumption was stronger than for any prelacteal feeding (crude – PR = 2.38; 95% CI = 2.12-2.68; adjusted – PR = 1.81; 95% CI = 1.67-1.97), and was not significant only in South Asia. Importantly, there was a significant association across all income groups, but especially in low and lower-middle income countries (Table S4 in the **Online Supplementary Document**). We found an inverse association between water-based only prelacteal feeds and exclusive breastfeeding with countries altogether (crude – PR = 0.67; 95% CI = 0.60-0.75; adjusted – PR = 0.69; 95% CI = 0.63-0.75) and across all income groups, but not for Eastern Europe and Central Asia, South Asia, and the Latin America and the Caribbean. For CMF consumption, we did not find an association with water-based only prelacteals in the pooled countries’ sample (crude – PR = 0.96; 95% CI = 0.86-1.08; adjusted – PR = 1.09; 95% CI = 0.99-1.20), nor the analysis by world region nor by income groups (Figures S4-S7 and Table S4 in the **Online Supplementary Document**). In Tables S5-S6 in the **Online Supplementary Document** we provide crude and adjusted estimates for the associations for each survey included in the analysis.

**Table 1** shows the percentage of different food types consumed by non-exclusively breastfed children according to the exposures milk-based and water-based only prelacteals by world regions. Children fed with milk-based only prelacteal early in life were more likely to be fed other milk, like formula or other animal milk, and less likely to have had plain water in all regions, except in South Asia. Additionally, in Eastern Europe and Central Asia, East Asia and Pacific, and Latin America and the Caribbean, the proportion of children still being breastfed during the first six months of life was lower for those exposed to milk-based only prelacteals. The patterns of associations for each country are shown in Table S7 in the **Online Supplementary Document**.

**Table 1:** Food types* consumed by non-exclusively breastfed children in the day before the survey by types of prelacteal, grouped by regions of the world and income groups

Groups	Any breastfeeding (%)		Plain water (%)		Other liquids (%)		Other milks (%)		Complementary foods (%)	
	**Milk-based prelacteals only**	**Water-based prelacteals only**	**Milk-based prelacteals only**	**Water-based prelacteals only**	**Milk-based prelacteals only**	**Water-based prelacteals only**	**Milk-based prelacteals only**	**Water-based prelacteals only**	**Milk-based prelacteals only**	**Water-based prelacteals only**
**World regions**										
West and Central Africa	98.7	97.8	52.5	69.9	18.6	12.3	28.2	13.4	20.8	21.6
Eastern and Southern Africa	92.3	98.1	46.0	51.3	16.4	17.9	38.9	11.3	20.0	28.3
Middle East and North Africa	85.6	89.0	51.9	57.4	18.2	21.4	58.0	39.4	22.1	25.7
Eastern Europe and Central Asia	75.1	82.3	61.5	73.0	29.9	32.9	55.0	35.6	21.0	16.6
South Asia	97.4	98.3	38.2	31.1	7.3	7.3	34.0	31.9	19.7	17.4
East Asia & Pacific	85.2	95.4	34.6	57.1	7.7	11.7	47.8	22.1	18.0	26.6
Latin America and Caribbean	86.9	91.6	52.9	61.7	22.8	25.1	63.3	45.7	20.9	30.6
**Income groups**										
Upper-middle income	82.0	85.7	58.9	72.1	24.2	27.4	61.9	47.5	20.0	28.6
Lower-middle income	89.2	95.6	43.3	51.5	16.9	15.9	45.5	22.5	19.1	22.1
Low income	97.9	98.6	50.8	64.1	15.8	14.2	27.8	12.6	23.1	23.3

*Food groups: other liquids (sugar water, juices, liquid soups/ clear broth, and other liquids), other milks (formula or animal milks (cow, goat, etc.)), and complementary foods (baby food, flesh, eggs, vegetables, fruits, yogurt and dairy, tubers and grains, and other solid-semisolid foods).

## DISCUSSION

In this multi-country retrospective study, we showed that the introduction of prelacteal feedings in the first three days of life affects later feeding practices among children under six months of age in LMICs. One-third of the sampled children were fed any prelacteal feedings and were 40% less likely to be exclusively breastfed; findings were consistent for types of prelacteal used. By contrast, CMF was directly associated with any and milk-based only prelacteals, but not with water-based only prelacteals. Our results varied by regions of the world and by income groups. The associations of CMF consumption with prelacteal feeding were stronger in East Asia and the Pacific and Eastern Europe and Central Asia compared to other regions, and in poor resource settings.

The negative impact of prelacteal feeds on breastfeeding outcomes has been previously documented in cohort studies and randomized controlled trials worldwide [30-35] and recently summarized in a meta-analysis [7]. The latter showed an inverse likelihood of being exclusively breastfed or receiving any breast milk among infants under six months of age who received prelacteal feeds [7]. We add substantially to the current evidence by confirming their external validity of these findings using nationally representative surveys from 85 LMICs.

Our findings have important implications for infant and young child feeding policymaking and advocacy globally. Exclusive breastfeeding rates have been increasing in the last decades, though not at the pace needed to meet the World Health Assembly target for 2030 of 70% of infants exclusively breastfed [10]. The negative relationship between prelacteal feeds and exclusively breastfeeding are crucial to investments in programmes and strategies that protect, support, and promote breastfeeding since birth.

When the onset of lactation is delayed, the risk of self-reported insufficient milk is increased, potentially leading to the increased feeding of CMF, reduced nursing frequency, and the decision to not breastfeed [36]. The Baby Friendly Hospital Initiative (BFHI) can help prevent the unnecessary introduction of prelacteal feeds by promoting immediate skin-to-skin contact and breastfeeding within the first hour after birth (step 4 of BFHI), which has a direct and positive effect on exclusive and any breastfeeding practices beyond the first months of life. Step 6 of BFHI recommends to “not provide breastfed newborns any food or fluids other than breast milk, unless medically indicated”, which is another key action necessary to reduce prelacteal feeding practices [4,37].

Nonetheless, many other additional factors shape women’s decision to breastfeed their offspring. Social influences and family support have been shown to strongly influence exclusive breastfeeding, as have peer-breastfeeding counselling for mothers and families, breast milk expression during work hours, and breastfeeding in public [38-40]. Likewise, maternity protection is critical when addressing breastfeeding willingness. Many women do not have paid maternity leave benefits of adequate length or do not have any all, especially women working in the informal labour market, showing the need for advancing social protection policies and programmes that can profoundly affect breastfeeding outcomes [41,42].

Structural barriers can lead women to choose CMF as an alternative feeding source when their decisions are strongly influenced by the absence of guaranteed rights or cultural and belief impediments. Although CMF is recognized as an adequate feeding source when babies cannot be breastfed, it hampers the proper establishment of breastfeeding by reducing nursing frequency, thus diminishing breast milk production [43]. In many countries, medically related conditions are cited to support the use of CMF when a child cannot be breastfed [7], even though such decisions could be biased by the influence of CMF companies in health facilities [44]. Grummer-Strawn et al. [45] found that 60% (68 out of 114) of paediatric associations globally were financially sponsored by companies that produce breast milk substitutes, especially in the Americas, Europe, and Asia. CMF companies strongly promote their products in hospital settings by offering free samples to new parents, discouraging them to feed their babies with breast milk. These practices undermine support and counselling on breastfeeding management, and unnecessary exposure to infant formula is a common practice during hospital staying [40,46,47]. Furthermore, aggressive CMF marketing has exponentially grown in the last decades worldwide, especially across emerging economies [8,9], with evidence existing violations of the International Code of Marketing of Breastmilk Substitutes [40].

We argue that unnecessary early exposure to CMF, especially milk-based prelacteals, during the first days after birth may discourage women from breastfeeding, during a sensitive time frame that is critical for the establishment of lactation [7,36]. We found that CMF consumption among infants under six months was only associated with milk-based prelacteals, but not with water-based only prelacteals. It is thus important to identify the main reasons associated with giving different types of prelacteal feeds. Milk-based prelacteals are often given when insufficient milk is self-reported or noted or due to medical conditions [2], while water-based prelacteals are commonly associated with biomedical (glucose water) and ritualistic reasons (small quantities or on a few occasions – normally prepared herbal-based mixtures) or the perception of baby’s thirst [2,48].

Regional differences must also be considered, as we found stronger associations of milk-based only prelacteals with CMF consumption in regions where CMF marketing has been steadily increasing recently, like East Asia and Pacific, Eastern Europe and Central Asia, and Latin America and Caribbean [8,9]. Additionally, our findings showed that in regions where the association between milk-based only prelacteals and CMF consumption was stronger, children not exclusively breastfed were more likely to consume other milks in the previous day compared to children in regions where the association was weaker. Also, children who received water-based only prelacteal and were not exclusively breastfed were more likely to consume plain water, other liquids, and complementary foods.

Integrated and novel policies, strategies, and interventions aimed at increasing breastfeeding rates must consider the constellation of modifiable factors that detrimentally influence lactation onset, such as caesarean section births and health care professional’s lack of training and knowledge about the onset and establishment of lactation [1,21,41]. As stated before, interventions should consider the reasons for giving different types of prelacteals in each context and world region, including the cultural beliefs leading to water-based prelacteal feeds and the behavioral and commercial influences behind milk-based prelacteals. Finally, our findings call for stronger protection, promotion, and support for breastfeeding since pregnancy, with strong regulation of CMF marketing prenatally and perinatally to prevent milk-based prelacteal feeding. This is unlikely to happen unless health providers are better trained on lactation support and on how baby behaviours influence caregivers' infant feeding choices [49]. Innovative research is needed to investigate encouraging interventions to address the unnecessary introduction of prelacteal feeds in the neonatal period [7].

Some limitations of our study are 1) its retrospective design which can introduce recall bias; however, we believe that this effect was minimal based on the consistency of our findings with a meta-analysis of prospective studies [7]; 2) some countries had to be excluded due to lack of convergence of the models, which could have affected the pooled estimates; 3) information for other types of prelacteal feeds is not available in DHS/MICS, like rice- or flour-based prelacteals [5]; however, milk-based and water-based prelacteal are most commonly used; 4) although the definition of prelacteal feeding used in national representative surveys differs from the WHO definition, it is the most pragmatic choice, as it best captures prelacteal feeding in large surveys.

The strengths of our analyses include: 1) the national-level representativeness of the data; 2) the geographical representation of the included countries; 3) the inclusion of quite diverse LMICs with highly comparable standardized surveys; and 4) examining the effects of different types of prelacteal feeds across countries and regions with very contrasting contexts.

## CONCLUSIONS

Our results contribute to the body of evidence highlighting the negative impact of prelacteal feeding practices on shortening exclusive breastfeeding duration and add new insights about the direct relationship between milk-based prelacteals and CMF consumption in LMICs. Pro-breastfeeding policies and interventions should consider the distinct effect that different types of prelacteals have on the outcomes studied in this analysis considering the regional, cultural, behavioural, and health professional training issues that drive the use of prelacteals. Women and members of their social support networks should receive breastfeeding education and lactation management counselling prenatally, perinatally, and throughout the hospital stay, and after discharge. This will require having well-trained health professionals and community health workers in all aspects of breastfeeding protection, promotion, and support.

## Additional material


Online Supplementary Document

## References

[1] WHO. Protecting, Promoting and Supporting Breastfeeding in Facilities Providing Maternity and Newborn Services. 2017. Available: https://apps.who.int/iris/bitstream/handle/10665/259386/9789241550086-eng.pdf?sequence=1&isAllowed=y. Accessed: 11 Nov 2022. 29565522

[2] McKenna KM Shankar RT The practice of prelacteal feeding to newborns among Hindu and Muslim families. J Midwifery Womens Health. 2009;54:78-81. . 10.1016/j.jmwh.2008.07.012 19114243

[3] Sundaram ME Labrique AB Mehra S Ali H Shamim AA Klemm RDW Early neonatal feeding is common and associated with subsequent breastfeeding behavior in rural Bangladesh. J Nutr. 2013;143:1161-7. . 10.3945/jn.112.170803 23677862

[4] UNICEF. From the first hour of life: Making the case for improved infant and young child feeding everywhere 2016:1–104. Available: https://data.unicef.org/resources/first-hour-life-new-report-breastfeeding-practices/. Accessed: 11 Nov 2022.

[5] Neves PAR Vaz JS Ricardo LIC Armenta-Paulino NN Barros AJD Richter L Disparities in early initiation of breast feeding and prelacteal feeding: A study of low- and middle-income countries. Paediatr Perinat Epidemiol. 2022;36:741-9. . 10.1111/ppe.12871 35253935

[6] Oakley L Benova L Macleod D Lynch CA Campbell OMR Early breastfeeding practices: Descriptive analysis of recent Demographic and Health Surveys. Matern Child Nutr. 2018;14:e12535. . 10.1111/mcn.12535 29034551 PMC5900960

[7] Pérez-Escamilla R Hromi-Fiedler A Rhodes EC Neves PAR Vaz J Vilar-Compte M Impact of prelacteal feeds and neonatal introduction of breast milk substitutes on breastfeeding outcomes: A systematic review and meta-analysis. Matern Child Nutr. 2022;18. . 10.1111/mcn.13368 35489107 PMC9113480

[8] Baker P Smith J Salmon L Friel S Kent G Iellamo A Global trends and patterns of commercial milk-based formula sales: Is an unprecedented infant and young child feeding transition underway? Public Health Nutr. 2016;19:2540-50. . 10.1017/S1368980016001117 27211798 PMC10270963

[9] Baker P Melo T Neves PA Machado P Smith J Piwoz E First-food systems transformations and the ultra-processing of infant and young child diets: The determinants, dynamics and consequences of the global rise in commercial milk formula consumption. Matern Child Nutr. 2021;17:e13097. . 10.1111/mcn.13097 33145965 PMC7988871

[10] Neves PAR Vaz JS Maia FS Baker P Gatica-Domínguez G Piwoz E Rates and time trends in the consumption of breastmilk, formula, and animal milk by children younger than 2 years from 2000 to 2019: analysis of 113 countries. Lancet Child Adolesc Health. 2021;5:619-30. . 10.1016/S2352-4642(21)00163-2 34245677 PMC8376656

[11] Victora CG Bahl R Barros AJD França GVA Horton S Krasevec J Breastfeeding in the 21st century: Epidemiology, mechanisms, and lifelong effect. Lancet. 2016;387:475-90. . 10.1016/S0140-6736(15)01024-7 26869575

[12] WHO/UNICEF. Global strategy for infant and young child feeding. Fifty-Fourth World Heal Assem 2003:1–30. Available: https://www.who.int/nutrition/publications/infantfeeding/9241562218/en/. Accessed: 11 Nov 2022.

[13] US Agency for International Development. Demographic and Health Surveys (DHS): what we do. Washington (DC): USAID; 2019. Available: https://dhsprogram.com/What-We-Do/index.cfm. Accessed: 10 Oct 2022.

[14] UNICEF. Multiple Indicator Cluster Surveys (MICS). New York (NY): UNICEF; 2019. Available: http://mics.unicef.org/. Accessed: 10 Oct 2022.

[15] National Institute of Statistics and Informatics. Encuesta Demográfica y de Salud Familiar. Lima, Peru: INEC. Available: https://proyectos.inei.gob.pe/endes/. Accessed: 10 Oct 2022.

[16] National Institute of Statistics. Encuesta Demográfica y de Salud 2016. La Paz, Bolivia: INE. Available: https://proyectos.inei.gob.pe/endes/. Accessed: 10 Oct 2022.

[17] International Center for Equity in Health. Surveys. Pelotas, Brazil: ICEH. Available: https://equidade.org/surveys. Accessed: 10 Oct 2022.

[18] Hancioglu A Arnold F Measuring coverage in MNCH: Tracking progress in health for women and children using DHS and MICS household surveys. PLoS Med. 2013;10. . 10.1371/journal.pmed.1001391 23667333 PMC3646216

[19] WHO. Indicators for assessing infant and young child feeding practices. Part 2 Measurement. World Heal Organ 2010:19. Available: https://apps.who.int/iris/bitstream/handle/10665/44306/9789241599290_eng.pdf?sequence=1. Accessed: 11 Nov 2022.

[20] Croft, TN, Aileen MJ. Marshall, Courtney K. Allen et al. Guide to DHS Statistics. DHS-7. Rockville, Maryland, USA ICF 2018:645.

[21] Boccolini CS Pérez-Escamilla R Giugliani ERJ Boccolini PDMM Inequities in milk-based prelacteal feedings in Latin America and the caribbean: The role of cesarean section delivery. J Hum Lact. 2015;31:89-98. . 10.1177/0890334414559074 25421875

[22] UNICEF. MICS6 Tools 2021. Available: https://mics.unicef.org/tools. Accessed: 14 Sep 2021.

[23] WHO/UNICEF. Indicators for assessing infant and young child feeding practices. Geneva: 2021. Available: https://www.who.int/publications/i/item/9789240018389. Accessed: 11 Nov 2022.

[24] Filmer D Pritchett LH Estimating Wealth Effects Without Expenditure Data - or Tears. Demography. 2001;38:115-32. . 10.1353/dem.2001.0003 11227840

[25] Rutstein S, Johnson K. The DHS wealth index. DHS Comparative Reports No. 6. 2004.

[26] Rutstein S. The DHS Wealth Index: Approaches for rural and urban areas. DHS Work Pap 2008.

[27] Johannes Textor. Directed Acyclic Graphs. Available: http://www.dagitty.net/manual-3.x.pdf. Accessed: 13 Nov 2022.

[28] Barros AJD Hirakata VN Alternatives for logistic regression in cross-sectional studies: na empirical comparison of models that directly estimate the prevalence ratio. BMC Med Res Methodol. 2003;3:21. . 10.1186/1471-2288-3-21 14567763 PMC521200

[29] UNICEF. UNICEF Regional classification 2019. Available: https://data.unicef.org/regionalclassifications/. Accessed: 30 Oct 2020.

[30] Forster DA McLachlan HL Lumley J Factors associated with breastfeeding at six months postpartum in a group of Australian women. Int Breastfeed J. 2006;1:18. . 10.1186/1746-4358-1-18 17034645 PMC1635041

[31] Bruun S Wedderkopp N Mølgaard C Kyhl HB Zachariassen G Husby S Using text messaging to obtain weekly data on infant feeding in a Danish birth cohort resulted in high participation rates. Acta Paediatr. 2016;105:648-54. . 10.1111/apa.13382 26928297

[32] Patil CL Turab A Ambikapathi R Nesamvuni C Chandyo RK Bose A Early interruption of exclusive breastfeeding: results from the eight-country MAL-ED study. J Health Popul Nutr. 2015;34:10. . 10.1186/s41043-015-0004-2 26825923 PMC5025973

[33] Balogun OO Kobayashi S Anigo KM Ota E Asakura K Sasaki S Factors Influencing Exclusive Breastfeeding in Early Infancy: A Prospective Study in North Central Nigeria. Matern Child Health J. 2016;20:363-75. . 10.1007/s10995-015-1835-6 26520155

[34] Chantry CJ Dewey K Peerson J Wagner E Nommsen-Rivers L In-hospital formula use increases early breastfeeding cessation among first-time mothers intending to exclusively breastfeed. J Pediatr. 2014;164:1339-45. e5. . 10.1016/j.jpeds.2013.12.035 24529621 PMC4120190

[35] Zakarija-Grković I Šegvić O Vučković Vukušić A Lozančić T Božinović T Ćuže A Predictors of suboptimal breastfeeding: an opportunity for public health interventions. Eur J Public Health. 2016;26:282-9. . 10.1093/eurpub/ckv203 26541859

[36] Segura-Pérez S Richter L Rhodes EC Hromi-Fiedler A Vilar-Compte M Adnew M Risk factors for self-reported insufficient milk during the first 6 months of life: A systematic review. Matern Child Nutr. 2022;18 Suppl 3:e13353. . 10.1111/mcn.13353 35343065 PMC9113468

[37] UNICEF/WHO. Capture the moment. Early initiation of breastfeeding: the best start for every newborn. New York: 2018. Available: https://www.unicef.org/eca/media/4256/file/Capture-the-moment-EIBF-report.pdf. Accessed: 11 Nov 2022.

[38] UNICEF. Children, food and nutrition: growing well in a changing world. 2019. Available: https://www.unicef.org/media/60806/file/SOWC-2019.pdf. Accessed: 11 Nov 2022.

[39] Li R Perrine CG Anstey EH Chen J Macgowan CA Elam-Evans LD Breastfeeding Trends by Race/Ethnicity among US Children Born from 2009 to 2015. JAMA Pediatr. 2019;173:e193319. . 10.1001/jamapediatrics.2019.3319 31609438 PMC6802058

[40] McFadden A Siebelt L Marshall JL Gavine A Girard LC Symon A Counselling interventions to enable women to initiate and continue breastfeeding: A systematic review and meta-analysis. Int Breastfeed J. 2019;14:42. . 10.1186/s13006-019-0235-8 31649743 PMC6805348

[41] Rollins NC Bhandari N Hajeebhoy N Horton S Lutter CK Martines JC Why invest, and what it will take to improve breastfeeding practices? Lancet. 2016;387:491-504. . 10.1016/S0140-6736(15)01044-2 26869576

[42] International Labour Organization. Maternity and paternity at work: law and practice across the world. Geneva: 2014. Available: https://www.ilo.org/wcmsp5/groups/public/@dgreports/@dcomm/@publ/documents/publication/wcms_242615.pdf. Accessed: 11 Nov 2022.

[43] Ministry of Health of Brazil. Dietary guidelines for Brazilian children under two years of age. 2019. Available: https://bvsms.saude.gov.br/bvs/publicacoes/dietary_guidelines_brazilian_chhildren_under.pdf. Accessed: 11 Nov 2022.

[44] WHO/UNICEF. How the marketing of formula milk influences our decisions on infant feeding. 2022. Available: https://www.who.int/publications/i/item/9789240044609. Accessed: 11 Nov 2022.

[45] Grummer-Strawn LM Holliday F Jungo KT Rollins N Sponsorship of national and regional professional paediatrics associations by companies that make breast-milk substitutes: Evidence from a review of official websites. BMJ Open. 2019;9:e029035. . 10.1136/bmjopen-2019-029035 31401600 PMC6701639

[46] Hastings G Angus K Eadie D Hunt K Selling second best: How infant formula marketing works. Global Health. 2020;16:77. . 10.1186/s12992-020-00597-w 32859218 PMC7455895

[47] Boccolini CS Victora CG Is there an “acceptable” percentage of using infant formula during hospital stays? J Pediatr (Rio J). 2022;98:439-41. . 10.1016/j.jped.2022.05.002 35644257 PMC9510797

[48] Akuse RM Obinya EA Why healthcare workers give prelacteal feeds. Eur J Clin Nutr. 2002;56:729-34. . 10.1038/sj.ejcn.1601385 12122548

[49] Vilar-Compte M Pérez-Escamilla R Orta-Aleman D Cruz-Villalba V Segura-Pérez S Nyhan K Impact of baby behaviour on caregiver’s infant feeding decisions during the first 6 months of life: A systematic review. Matern Child Nutr. 2022;18 Suppl 3 e13345. . 10.1111/mcn.13345 35363420 PMC9113474

