# Supplementary material for: Prelacteal feeding and its relationship with exclusive breastfeeding and formula consumption among infants in low- and middle-income countries
Source: J Glob Health. 2022 Dec 23;12:04104. doi: 10.7189/jogh.12.04104 (PMC9789363; doi:10.7189/jogh.12.04104)
Supplement: Online Supplementary Document [file jogh-12-04104-s001.pdf]

Table S1. Description of the countries included in the analysis.

ISO	Country	Year of the survey	Regions of the world	Income group (2015)
AFG	Afghanistan	2015	South Asia	Low income
DZA	Algeria	2018	Middle East & North Africa	Upper-middle income
AGO	Angola	2015	Eastern & Southern Africa	Upper-middle income
ARM	Armenia	2010	Eastern Europe & Central Asia	Lower-middle income
BGD	Bangladesh	2019	South Asia	Lower-middle income
BLR	Belarus	2012	Eastern Europe & Central Asia	Upper-middle income
BLZ	Belize	2015	Latin America & Caribbean	Upper-middle income
BEN	Benin	2014	West & Central Africa	Low income
BTN	Bhutan	2010	South Asia	Lower-middle income
BOL	Bolivia	2016	Latin America & Caribbean	Lower-middle income
BFA	Burkina Faso	2010	West & Central Africa	Low income
CAF	Central African Republic	2018	West & Central Africa	Low income
KHM	Cambodia	2014	East Asia & Pacific	Lower-middle income
CMR	Cameroon	2014	West & Central Africa	Lower-middle income
TCD	Chad	2019	West & Central Africa	Low income
COL	Colombia	2010	Latin America & Caribbean	Upper-middle income
COM	Comoros	2012	Eastern & Southern Africa	Low income
COG	Congo Brazzaville	2014	West & Central Africa	Lower-middle income
COD	Congo Democratic Republic	2017	West & Central Africa	Low income
CRI	Costa Rica	2018	Latin America & Caribbean	Upper-middle income
CIV	Cote d’Ivoire	2016	West & Central Africa	Lower-middle income
CUB	Cuba	2019	Latin America & Caribbean	Upper-middle income
DOM	Dominican Republic	2014	Latin America & Caribbean	Upper-middle income
EGY	Egypt	2014	Middle East & North Africa	Lower-middle income
SLV	El Salvador	2014	Latin America & Caribbean	Lower-middle income
SWZ	Eswatini	2014	Eastern & Southern Africa	Lower-middle income
ETH	Ethiopia	2011	Eastern & Southern Africa	Low income
GAB	Gabon	2012	West & Central Africa	Upper-middle income
GMB	Gambia	2018	West & Central Africa	Low income
GHA	Ghana	2017	West & Central Africa	Lower-middle income
GTM	Guatemala	2014	Latin America & Caribbean	Lower-middle income
GIN	Guinea	2016	West & Central Africa	Low income
GNB	Guinea Bissau	2018	West & Central Africa	Low income
GUY	Guyana	2014	Latin America & Caribbean	Upper-middle income
HTI	Haiti	2012	Latin America & Caribbean	Low income
HND	Honduras	2011	Latin America & Caribbean	Lower-middle income
IND	India	2015	South Asia	Lower-middle income
IDN	Indonesia	2017	East Asia & Pacific	Lower-middle income
IRQ	Iraq	2018	Middle East & North Africa	Upper-middle income
JAM	Jamaica	2011	Latin America & Caribbean	Upper-middle income
JOR	Jordan	2017	Middle East & North Africa	Upper-middle income
KAZ	Kazakhstan	2015	Eastern Europe & Central Asia	Upper-middle income
KIR	Kiribati	2018	East Asia & Pacific	Lower-middle income
XKX	Kosovo	2019	Eastern Europe & Central Asia	Lower-middle income
KGZ	Kyrgyzstan	2018	Eastern Europe & Central Asia	Lower-middle income

ISO	Country	Year of the survey	Regions of the world	Income group (2015)
LAO	Lao	2017	East Asia & Pacific	Lower-middle income
LSO	Lesotho	2018	Eastern & Southern Africa	Lower-middle income
LBR	Liberia	2013	West & Central Africa	Low income
MDG	Madagascar	2018	Eastern & Southern Africa	Low income
MLI	Mali	2015	West & Central Africa	Low income
MRT	Mauritania	2015	West & Central Africa	Lower-middle income
MEX	Mexico	2015	Latin America & Caribbean	Upper-middle income
MDA	Moldova	2012	Eastern Europe & Central Asia	Lower-middle income
MNG	Mongolia	2018	East Asia & Pacific	Lower-middle income
MNE	Montenegro	2018	Eastern Europe & Central Asia	Upper-middle income
MOZ	Mozambique	2011	Eastern & Southern Africa	Low income
MMR	Myanmar	2015	East Asia & Pacific	Lower-middle income
NAM	Namibia	2013	Eastern & Southern Africa	Upper-middle income
NPL	Nepal	2019	South Asia	Low income
NER	Niger	2012	West & Central Africa	Low income
NGA	Nigeria	2016	West & Central Africa	Lower-middle income
MKD	North Macedonia	2018	Eastern Europe & Central Asia	Upper-middle income
PAK	Pakistan	2012	South Asia	Lower-middle income
PAN	Panama	2013	Latin America & Caribbean	Upper-middle income
PRY	Paraguay	2016	Latin America & Caribbean	Upper-middle income
PER	Peru	2019	Latin America & Caribbean	Upper-middle income
STP	Sao Tome and Principe	2019	West & Central Africa	Lower-middle income
SEN	Senegal	2019	West & Central Africa	Low income
SRB	Serbia	2019	Eastern Europe & Central Asia	Upper-middle income
SLE	Sierra Leone	2017	West & Central Africa	Low income
PSE	State of Palestine	2019	Middle East & North Africa	Lower-middle income
SDN	Sudan	2014	Eastern & Southern Africa	Lower-middle income
SUR	Suriname	2018	Latin America & Caribbean	Upper-middle income
TJK	Tajikistan	2012	Eastern Europe & Central Asia	Lower-middle income
TZA	Tanzania	2010	Eastern & Southern Africa	Low income
THA	Thailand	2019	East Asia & Pacific	Upper-middle income
TGO	Togo	2017	West & Central Africa	Low income
TON	Tonga	2019	East Asia & Pacific	Lower-middle income
TUN	Tunisia	2018	Middle East & North Africa	Lower-middle income
TUR	Turkey	2013	Eastern Europe & Central Asia	Upper-middle income
UKR	Ukraine	2012	Eastern Europe & Central Asia	Lower-middle income
VNM	Vietnam	2013	East Asia & Pacific	Lower-middle income
YEM	Yemen	2013	Middle East & North Africa	Lower-middle income
ZMB	Zambia	2013	Eastern & Southern Africa	Lower-middle income
ZWE	Zimbabwe	2019	Eastern & Southern Africa	Low income

Table S2. Countries and prevalence of the feeding indicators. Source: Demographic Health Survey, Multiple Indicator Cluster Survey, *Encuesta Demográfica y de Salud Familiar*, and *Encuesta Demográfica y de Salud*, 2010-2019.

Country	Year	Exclusive breastfeeding under six months (%)			Formula consumption under six months (%)			Any prelacteal feeding (%)			Milk-based only prelacteal feeding (%)			Water-based only prelacteal feeding (%)			Number of children
		Prevalence	95% CI		Prevalence	95% CI		Prevalence	95% CI		Prevalence	95% CI		Prevalence	95% CI		
All LMICs*	-	35.2	34.9	35.5	27.7	27.4	28.0	33.9	33.6	34.2	22.2	21.9	22.4	9.4	9.2	9.6	91282
Afghanistan	2015	43.6	41.9	45.4	8.4	7.4	9.3	40.5	38.8	42.2	2.5	2.0	3.1	34.8	33.2	36.5	3181
Algeria	2018	32.4	29.9	35.0	30.4	27.9	32.9	52.5	49.8	55.2	15.5	13.5	17.5	26.6	24.2	29.0	1303
Angola	2015	39.8	37.4	42.3	7.1	5.8	8.4	11.0	9.4	12.5	4.0	3.0	5.0	6.7	5.5	8.0	1531
Armenia	2010	34.6	27.1	42.1	15.6	9.9	21.3	13.5	8.1	18.8	7.7	3.5	11.9	5.8	2.1	9.4	156
Bangladesh	2019	63.4	61.4	65.3	13.3	11.9	14.6	23.7	22.0	25.4	11.4	10.1	12.7	9.9	8.7	11.1	2355
Belarus	2012	20.5	15.3	25.8	46.3	39.8	52.7	44.5	38.0	51.0	31.8	25.8	37.9	6.8	3.5	10.0	228
Belize	2015	32.2	24.9	39.5	41.8	34.1	49.6	15.9	10.2	21.6	8.0	3.8	12.3	4.0	0.9	7.1	157
Benin	2014	41.4	38.7	44.1	1.2	0.6	1.8	21.4	19.1	23.7	1.6	0.9	2.3	18.9	16.7	21.1	1249
Bhutan	2010	49.6	45.5	53.7	6.8	4.7	8.8	7.3	5.2	9.5	4.0	2.4	5.7	2.1	0.9	3.3	580
Bolivia	2016	60.2	55.6	64.8	23.6	19.6	27.6	27.9	23.7	32.1	20.4	16.6	24.1	6.6	4.3	8.9	440
Burkina Faso	2010	24.8	22.1	27.8	0.8	0.4	1.5	32.7	29.6	36.0	0.6	0.2	1.0	31.9	29.5	34.3	1452
Cambodia	2014	66.9	63.3	70.5	9.6	7.3	11.8	29.2	25.7	32.6	11.9	9.4	14.3	9.2	7.0	11.4	667
Cameroon	2014	29.0	25.5	32.4	7.8	5.8	9.8	44.6	40.9	48.4	1.4	0.5	2.2	41.8	38.1	45.6	679
Central African Republic	2018	37.9	34.8	41.1	3.0	1.9	4.1	10.9	8.9	12.9	2.0	1.1	2.9	7.9	6.1	9.6	928
Chad	2014	0.3	0.0	0.5	4.5	3.5	5.5	86.8	85.2	88.3	2.8	2.1	3.6	71.3	69.2	73.4	1813
Colombia	2010	43.3	40.8	45.8	35.0	32.6	37.5	37.1	34.6	39.5	29.3	27.0	31.6	6.2	5.0	7.4	1498
Comoros	2012	12.4	8.8	16.1	26.4	21.5	31.2	39.5	34.2	44.9	7.3	4.4	10.1	31.4	26.3	36.5	318
Congo Brazzaville	2014	33.7	30.5	36.8	9.9	7.9	11.9	31.4	28.2	34.5	15.3	12.9	17.8	12.7	10.5	15.0	846
CDR**	2017	55.0	52.9	57.2	5.4	4.4	6.3	9.1	7.8	10.3	1.6	1.0	2.1	5.7	4.7	6.6	2066
Costa Rica	2018	27.2	21.8	32.5	41.2	35.3	47.2	43.3	37.3	49.3	42.6	36.6	48.6	0.2	0.0	0.7	263
Cote d'Ivoire	2016	23.4	20.7	26.1	5.1	3.7	6.5	41.4	38.3	44.5	2.5	1.5	3.5	37.8	34.7	40.9	954
Cuba	2019	43.9	32.1	56.3	8.4	4.0	17.0	8.1	4.3	14.7	7.3	4.1	10.4	0.5	0.0	1.3	259
Dominican Republic	2014	4.9	3.8	6.0	30.8	28.4	33.1	57.6	55.1	60.1	52.3	49.8	54.8	2.4	1.7	3.2	1526
Egypt	2014	41.2	38.7	43.7	13.3	11.5	15.0	63.4	60.9	65.9	6.5	5.2	7.8	53.5	50.9	56.0	1437
El Salvador	2014	47.8	43.4	52.2	30.4	26.3	34.4	26.3	22.4	30.1	24.7	20.9	28.4	1.6	0.5	2.7	497

Country	Year	Exclusive breastfeeding under six months (%)			Formula consumption under six months (%)			Any prelacteal feeding (%)			Milk-based only prelacteal feeding (%)			Water-based only prelacteal feeding (%)			Number of children
		Prevalence	95% CI		Prevalence	95% CI		Prevalence	95% CI		Prevalence	95% CI		Prevalence	95% CI		
Eswatini	2014	68.4	62.2	74.7	10.1	6.1	14.2	14.2	9.5	18.9	0.0	0.0	0.0	13.4	8.8	18.0	214
Ethiopia	2011	52.3	49.4	55.1	2.6	1.7	3.5	28.4	25.9	31.0	6.0	4.6	7.4	21.5	19.1	23.8	1178
Gabon	2012	6.3	4.4	8.3	63.5	59.7	67.4	42.0	38.0	45.9	20.6	17.3	23.8	15.4	12.5	18.2	598
Gambia	2018	55.1	51.8	58.3	5.0	3.6	6.5	10.3	8.3	12.3	0.5	0.0	1.0	9.4	7.5	11.3	900
Ghana	2017	42.5	39.2	45.8	17.6	15.1	20.2	15.3	12.9	17.7	3.2	2.1	4.4	8.4	6.5	10.2	866
Guatemala	2014	54.3	51.4	57.2	19.6	17.3	21.9	34.0	31.2	36.7	24.7	22.2	27.2	7.8	6.2	9.3	1152
Guinea	2016	35.4	31.7	39.0	6.8	4.9	8.8	44.0	40.2	47.8	1.9	0.9	3.0	41.8	38.0	45.6	656
Guinea Bissau	2018	62.6	58.9	66.2	2.7	1.5	3.9	12.8	10.3	15.3	0.2	0.0	0.5	12.1	9.7	14.6	678
Guyana	2014	24.8	19.5	30.1	42.3	36.2	48.3	21.4	16.4	26.4	14.3	10.1	18.6	4.4	1.9	6.9	257
Haiti	2012	40.5	36.9	44.1	17.3	14.6	20.1	20.1	17.2	23.1	1.8	0.8	2.7	17.1	14.4	19.9	715
Honduras	2011	31.8	29.0	34.6	25.6	23.0	28.2	44.1	41.1	47.0	25.5	22.9	28.1	12.9	10.8	14.9	1065
India	2015	56.2	55.5	56.8	3.6	3.4	3.8	20.4	19.9	20.9	11.7	11.3	12.1	6.1	5.8	6.4	22054
Indonesia	2017	53.3	50.8	55.7	26.9	24.7	29.0	45.0	42.6	47.4	36.8	34.4	39.1	5.3	4.2	6.4	1615
Iraq	2018	28.2	25.9	30.4	42.6	40.1	45.0	52.5	50.0	55.0	24.7	22.5	26.8	19.4	17.4	21.3	1583
Jamaica	2011	24.8	18.1	31.6	50.8	43.0	58.5	23.6	17.0	30.2	21.0	14.6	27.3	1.6	0.0	3.5	160
Jordan	2017	27.3	24.7	29.9	48.2	45.3	51.2	43.1	40.2	46.0	30.2	27.6	32.9	6.8	5.3	8.3	1129
Kazakhstan	2015	38.4	34.1	42.6	19.1	15.6	22.6	14.7	11.6	17.8	10.0	7.3	12.6	2.5	1.1	3.9	497
Kiribati	2018	69.1	62.9	75.2	11.7	7.5	16.0	25.8	20.1	31.6	5.8	2.7	8.9	16.9	12.0	21.9	221
Kosovo	2019	30.4	22.8	38.1	38.4	30.2	46.5	42.9	34.7	51.2	40.3	32.2	48.5	2.1	0.0	4.4	139
Kyrgyzstan	2018	46.9	41.9	51.8	12.7	9.4	16.1	12.4	9.1	15.7	7.9	5.2	10.5	3.8	1.9	5.7	387
Lao	2017	46.9	43.9	49.9	13.9	11.8	15.9	23.4	20.9	25.9	10.1	8.3	11.9	10.4	8.6	12.2	1088
Lesotho	2018	60.8	54.1	67.5	17.3	12.1	22.5	14.1	9.3	18.8	5.9	2.6	9.1	5.0	2.0	8.0	204
Liberia	2013	55.7	52.0	59.3	4.4	2.9	5.9	10.7	8.5	13.0	0.1	0.0	0.4	10.5	8.3	12.8	714
Madagascar	2018	51.4	48.8	54.1	2.2	1.4	3.0	34.3	31.8	36.8	3.4	2.4	4.4	30.2	27.7	32.6	1349
Mali	2015	32.4	30.1	34.7	1.4	0.8	2.0	27.5	25.3	29.7	3.8	2.8	4.7	20.7	18.7	22.7	1586
Mauritania	2015	41.8	38.5	45.1	9.8	7.8	11.8	28.9	25.9	31.9	9.7	7.7	11.7	15.4	13.0	17.8	853
Mexico	2015	32.4	28.8	36.1	46.5	42.6	50.4	35.2	31.5	38.9	32.9	29.2	36.6	1.7	0.7	2.7	629
Moldova	2012	37.7	30.4	44.9	21.6	15.4	27.7	27.3	20.6	34.0	19.2	13.3	25.1	6.5	2.8	10.2	172
Mongolia	2018	51.4	47.4	55.4	20.8	17.6	24.1	21.5	18.2	24.8	15.7	12.8	18.6	4.4	2.8	6.0	601

Country	Year	Exclusive breastfeeding under six months (%)			Formula consumption under six months (%)			Any prelacteal feeding (%)			Milk-based only prelacteal feeding (%)			Water-based only prelacteal feeding (%)			Number of children
		Prevalence	95% CI		Prevalence	95% CI		Prevalence	95% CI		Prevalence	95% CI		Prevalence	95% CI		
Montenegro	2018	23.5	15.0	32.1	34.1	24.5	43.7	36.5	26.8	46.3	35.5	25.9	45.2	0.0	0.0	0.0	95
Mozambique	2011	41.6	38.6	44.6	3.9	2.7	5.1	6.1	4.6	7.5	1.3	0.6	2.0	4.6	3.3	5.9	1028
Myanmar	2015	51.5	47.0	56.1	5.8	3.7	7.9	20.9	17.2	24.6	15.4	12.1	18.7	5.3	3.3	7.4	465
Namibia	2013	49.9	45.6	54.3	13.8	10.8	16.8	12.4	9.5	15.3	6.5	4.3	8.6	4.8	2.9	6.7	509
Nepal	2019	61.9	57.6	66.2	4.4	2.6	6.2	14.9	11.7	18.0	13.5	10.5	16.5	0.4	0.0	1.0	491
Niger	2012	23.3	21.0	25.6	1.2	0.6	1.8	48.0	45.3	50.7	11.6	9.8	13.3	33.2	30.7	35.8	1299
Nigeria	2016	24.0	22.4	25.7	6.1	5.2	7.0	52.7	50.8	54.6	2.7	2.1	3.4	44.7	42.8	46.7	2601
North Macedonia	2018	19.5	11.8	27.1	32.5	23.4	41.5	62.3	52.9	71.7	57.1	47.6	66.7	0.3	0.0	1.3	104
Pakistan	2012	38.7	35.8	41.6	8.9	7.1	10.6	75.1	72.5	77.7	27.2	24.5	29.9	29.2	26.5	32.0	1055
Panama	2013	21.4	17.8	25.1	54.7	50.3	59.2	33.7	29.5	37.9	31.3	27.1	35.4	1.0	0.1	1.9	482
Paraguay	2016	31.9	27.2	36.6	27.6	23.1	32.1	30.7	26.1	35.4	24.7	20.4	29.1	3.5	1.7	5.4	379
Peru	2019	65.6	63.3	67.9	18.8	16.8	20.7	40.9	38.4	43.3	40.2	37.8	42.6	0.4	0.1	0.7	1591
Sao Tome and Principe	2019	64.3	57.2	71.4	5.4	2.0	8.7	10.8	6.2	15.4	1.1	0.0	2.6	9.2	4.9	13.5	176
Senegal	2019	41.2	37.3	45.1	6.8	4.8	8.8	55.2	51.3	59.2	12.3	9.7	14.9	36.1	32.3	39.9	611
Serbia	2019	26.7	17.2	36.2	30.9	20.9	40.8	65.3	55.0	75.5	43.9	33.2	54.5	10.0	3.6	16.5	84
Sierra Leone	2017	53.0	50.1	55.9	8.8	7.2	10.5	7.7	6.2	9.3	0.9	0.3	1.4	6.6	5.1	8.0	1133
State of Palestine	2019	40.8	37.0	44.6	34.1	30.5	37.8	41.6	37.8	45.4	31.7	28.1	35.3	7.2	5.2	9.2	645
Sudan	2014	55.8	53.3	58.3	3.1	2.2	4.0	28.7	26.4	31.1	2.6	1.8	3.4	24.3	22.1	26.4	1469
Suriname	2018	9.6	6.3	13.0	56.5	50.9	62.0	36.7	31.3	42.1	32.6	27.3	37.9	1.7	0.3	3.2	304
Tajikistan	2012	34.3	29.8	38.9	11.4	8.3	14.4	12.6	9.4	15.7	0.8	0.0	1.6	10.7	7.7	13.6	423
Tanzania	2010	50.7	46.4	55.0	1.0	0.4	2.2	30.0	26.3	33.8	5.3	3.7	6.9	23.5	20.6	26.5	791
Thailand	2019	18.6	15.6	21.6	42.2	38.4	46.1	20.8	17.6	23.9	13.7	11.1	16.4	5.8	4.0	7.6	638
Togo	2017	65.5	61.4	69.7	2.7	1.3	4.2	13.2	10.2	16.2	1.9	0.7	3.1	6.4	4.3	8.6	500
Tonga	2019	46.2	36.6	55.9	38.9	29.5	48.3	16.8	9.6	24.1	14.7	7.9	21.5	1.3	0.0	3.4	104
Tunisia	2018	14.6	10.4	18.8	49.1	43.1	55.0	41.5	35.7	47.4	27.0	21.8	32.3	7.1	4.0	10.1	273
Turkey	2013	31.1	26.1	36.1	31.0	25.9	36.0	39.3	34.0	44.5	30.7	25.7	35.7	6.9	4.1	9.6	329
Ukraine	2012	19.2	13.4	26.8	29.8	22.3	38.6	11.4	7.0	18.1	2.5	0.7	4.2	7.3	4.3	10.3	293
Vietnam	2013	24.8	20.2	29.3	36.0	30.9	41.0	78.8	74.5	83.1	49.7	44.4	54.9	5.6	3.1	8.0	347
Yemen	2013	10.4	8.9	11.9	32.2	29.9	34.4	68.7	66.4	70.9	10.2	8.7	11.7	44.3	41.9	46.8	1645

Country	Year	Exclusive breastfeeding under six months (%)			Formula consumption under six months (%)			Any prelacteal feeding (%)			Milk-based only prelacteal feeding (%)			Water-based only prelacteal feeding (%)			Number of children
		Prevalence	95% CI		Prevalence	95% CI		Prevalence	95% CI		Prevalence	95% CI		Prevalence	95% CI		
Zambia	2013	72.5	69.9	75.0	1.5	0.8	2.2	5.0	3.8	6.3	1.5	0.8	2.2	3.4	2.4	4.5	1176
Zimbabwe	2019	42.3	38.4	46.3	3.7	2.2	5.2	12.7	10.0	15.4	1.7	0.7	2.8	4.7	3.0	6.3	599

*LMICs- low- and middle-income countries; **CDR – Congo Democratic Republic

Table S3. Exclusive breastfeeding and formula consumption prevalence by types of prelacteal feeding.

Country	Child received prelacteal feed	Any prelacteal feeding		Milk-based only prelacteal feeding		Water-based only prelacteal feeding	
		Exclusive breastfeeding (%)	Formula consumption (%)	Exclusive breastfeeding (%)	Formula consumption (%)	Exclusive breastfeeding (%)	Formula consumption (%)
All LMICs*	No	43.5	19.5	40.7	19.1	36.2	29.3
	Yes	19.0	43.6	15.8	57.7	25.8	11.9
Afghanistan	No	46.9	7.0	43.9	8.2	45.5	8.4
	Yes	38.9	10.4	33.8	15.5	40.2	8.3
Algeria	No	42.4	19.9	32.8	27.9	36.0	28.5
	Yes	23.4	39.9	30.2	43.9	22.4	35.8
Angola	No	42.9	5.1	40.9	5.3	41.5	7.3
	Yes	15.1	23.0	14.3	51.2	16.0	4.3
Armenia	No	36.2	16.2	35.7	15.9	35.0	15.9
	Yes	24.2	11.7	21.2	11.9	28.3	11.4
Bangladesh	No	67.8	9.6	65.6	10.9	64.1	13.1
	Yes	49.1	25.1	45.6	31.4	56.9	14.8
Belarus	No	30.7	37.9	25.3	43.1	22.0	44.5
	Yes	7.8	56.7	10.3	53.1	0.0	71.1
Belize	No	35.4	37.2	34.2	38.7	33.1	41.8
	Yes	15.4	66.1	9.6	77.5	10.7	41.6
Benin	No	47.0	1.0	41.8	1.2	45.8	1.0
	Yes	20.8	1.9	14.1	3.6	22.3	1.9
Bhutan	No	50.4	7.0	50.2	6.7	49.4	6.9
	Yes	38.8	4.2	34.4	7.6	59.4	0.0
Bolivia	No	69.8	14.3	66.5	16.2	61.8	23.4
	Yes	35.5	47.6	35.5	52.6	38.3	26.8
Burkina Faso	No	34.5	0.6	24.9	0.7	34.2	0.9
	Yes	5.0	1.1	16.0	11.3	4.8	0.4

Country	Child received prelacteal feed	Any prelacteal feeding		Milk-based only prelacteal feeding		Water-based only prelacteal feeding	
		Exclusive breastfeeding (%)	Formula consumption (%)	Exclusive breastfeeding (%)	Formula consumption (%)	Exclusive breastfeeding (%)	Formula consumption (%)
Central African Republic	No	39.7	2.6	38.0	2.6	39.5	3.0
	Yes	23.4	6.4	32.3	24.4	19.2	2.7
Cambodia	No	74.0	5.4	68.8	7.3	68.4	9.9
	Yes	49.7	19.7	53.1	26.1	51.9	5.8
Cameroon	No	42.0	7.8	29.1	7.3	41.1	8.7
	Yes	12.7	7.9	17.5	46.5	12.0	6.6
Chad	No	0.3	5.2	0.3	4.1	0.4	8.7
	Yes	0.3	4.4	0.4	16.4	0.3	2.8
Colombia	No	50.5	28.2	47.7	28.5	44.3	35.8
	Yes	30.9	46.7	32.6	50.9	27.6	23.0
Comoros	No	17.2	27.2	11.8	25.6	17.3	28.8
	Yes	5.2	25.0	20.0	36.6	1.9	21.1
Congo Brazzaville	No	34.0	8.9	32.6	7.9	35.0	10.6
	Yes	33.0	11.9	39.7	20.5	24.8	4.6
Congo Democratic Republic	No	55.8	4.6	54.8	5.1	56.0	4.9
	Yes	47.5	13.2	68.7	21.3	39.3	14.1
Costa Rica	No	39.4	28.1	39.6	28.2	27.2	41.2
	Yes	11.1	58.5	10.4	58.9	0.0	47.4
Cote d'Ivoire	No	31.9	3.5	23.6	4.2	31.0	5.2
	Yes	11.4	7.4	16.7	41.8	11.0	5.0
Cuba	No	45.6	8.0	45.3	7.9	44.0	8.5
	Yes	23.7	13.9	25.0	15.5	21.0	0.0
Dominican Republic	No	7.6	24.8	7.0	26.5	5.0	30.4
	Yes	2.9	35.1	3.0	34.6	2.5	43.4
Egypt	No	45.5	8.8	41.7	12.1	42.1	13.9
	Yes	38.7	15.8	34.5	30.1	40.4	12.7

Country	Child received prelacteal feed	Any prelacteal feeding		Milk-based only prelacteal feeding		Water-based only prelacteal feeding	
		Exclusive breastfeeding (%)	Formula consumption (%)	Exclusive breastfeeding (%)	Formula consumption (%)	Exclusive breastfeeding (%)	Formula consumption (%)
El Salvador	No	54.2	21.3	53.7	21.9	48.1	30.1
	Yes	29.6	55.8	29.7	56.3	28.4	47.1
Eswatini	No	72.2	10.3	68.4	10.1	71.5	10.2
	Yes	45.6	9.4	0.0	0.0	48.3	9.9
Ethiopia	No	53.6	2.7	54.9	2.5	50.1	2.8
	Yes	48.8	2.4	10.8	4.7	60.1	1.9
Gabon	No	8.6	55.1	7.3	57.9	6.6	64.0
	Yes	3.2	75.2	2.4	85.1	5.1	61.0
Gambia	No	57.2	4.6	55.1	4.9	57.3	4.7
	Yes	36.4	8.7	41.6	26.2	33.3	8.1
Ghana	No	47.3	15.4	43.0	17.4	45.3	16.8
	Yes	15.6	30.2	27.3	25.0	12.1	27.2
Guatemala	No	66.1	7.5	62.6	9.0	55.3	20.3
	Yes	31.3	43.1	28.9	51.9	42.3	11.7
Guinea	No	43.4	6.6	35.5	6.7	42.9	7.1
	Yes	25.1	7.2	27.0	16.4	24.8	6.5
Guinea Bissau	No	63.7	2.0	62.7	2.7	63.5	2.3
	Yes	54.9	7.3	0.0	0.0	55.7	5.9
Guyana	No	27.6	37.9	27.3	37.7	24.7	43.5
	Yes	14.6	58.1	10.1	69.3	28.3	15.4
Haiti	No	46.0	15.4	41.1	16.6	44.7	16.7
	Yes	18.9	24.9	9.7	60.3	20.2	20.6
Honduras	No	39.8	16.6	35.8	18.5	31.8	26.8
	Yes	21.6	37.1	20.0	46.3	31.5	17.6
India	No	59.5	3.6	57.9	3.6	56.9	3.6
	Yes	43.4	3.7	43.4	3.8	45.8	3.7

Country	Child received prelacteal feed	Any prelacteal feeding		Milk-based only prelacteal feeding		Water-based only prelacteal feeding	
		Exclusive breastfeeding (%)	Formula consumption (%)	Exclusive breastfeeding (%)	Formula consumption (%)	Exclusive breastfeeding (%)	Formula consumption (%)
Indonesia	No	62.4	15.7	61.3	16.6	53.1	27.8
	Yes	42.0	40.4	39.5	44.5	56.5	9.9
Iraq	No	37.4	31.9	32.0	34.2	28.4	46.1
	Yes	19.8	52.2	16.4	68.1	27.3	27.6
Jamaica	No	26.5	50.4	26.2	50.5	24.8	50.4
	Yes	19.4	52.0	19.8	51.7	27.6	72.4
Jordan	No	32.9	39.3	30.5	41.0	28.0	48.5
	Yes	19.8	60.0	19.9	65.0	17.5	44.3
Kazakhstan	No	42.5	15.6	40.6	16.3	39.2	19.0
	Yes	14.5	39.7	18.2	44.1	7.0	23.7
Kiribati	No	72.1	9.0	70.4	9.8	69.9	11.7
	Yes	60.4	19.7	46.7	42.7	64.9	11.9
Kosovo	No	37.4	21.9	37.8	21.9	29.9	39.2
	Yes	21.2	60.2	19.6	62.7	57.8	0.0
Kyrgyzstan	No	47.7	11.0	47.2	11.7	47.0	12.8
	Yes	40.6	25.1	42.5	25.4	42.0	12.3
Lao	No	54.5	9.1	49.2	10.9	49.9	13.9
	Yes	22.1	29.7	26.5	40.8	21.1	13.9
Lesotho	No	61.7	13.9	62.5	14.6	61.3	16.2
	Yes	55.1	38.0	33.3	60.1	51.5	36.7
Liberia	No	59.7	4.6	55.6	4.4	59.7	4.6
	Yes	21.8	2.8	100.0	0.0	21.1	2.8
Madagascar	No	59.4	1.6	51.6	1.7	58.4	2.4
	Yes	36.2	3.4	45.7	16.9	35.3	1.8
Mali	No	36.7	1.5	32.7	1.4	35.4	1.6
	Yes	21.2	1.2	25.3	2.4	20.9	0.9

Country	Child received prelacteal feed	Any prelacteal feeding		Milk-based only prelacteal feeding		Water-based only prelacteal feeding	
		Exclusive breastfeeding (%)	Formula consumption (%)	Exclusive breastfeeding (%)	Formula consumption (%)	Exclusive breastfeeding (%)	Formula consumption (%)
Mauritania	No	47.9	8.4	43.1	8.7	44.6	9.7
	Yes	26.9	13.1	29.6	19.3	26.4	10.2
Mexico	No	39.6	36.3	38.5	36.5	32.9	46.9
	Yes	19.2	65.3	20.1	66.8	7.1	23.7
Moldova	No	42.5	16.0	43.0	15.3	37.5	22.3
	Yes	24.9	36.3	15.4	47.9	39.4	11.1
Mongolia	No	55.2	16.5	55.4	16.9	51.3	20.8
	Yes	37.4	36.8	29.9	42.2	52.3	21.7
Montenegro	No	18.6	33.0	19.8	32.5	23.5	34.1
	Yes	32.1	36.0	30.2	37.0	-	-
Mozambique	No	42.6	3.6	41.7	3.7	42.6	3.8
	Yes	25.4	8.8	32.9	18.3	21.3	6.4
Myanmar	No	55.2	3.0	52.9	3.3	53.5	5.7
	Yes	37.8	16.3	44.1	19.5	17.1	7.7
Namibia	No	53.3	12.8	51.9	12.3	50.5	14.2
	Yes	26.2	20.7	20.9	34.4	37.8	4.5
Nepal	No	64.2	2.9	64.2	3.3	62.0	4.3
	Yes	48.7	13.0	47.1	11.7	46.1	39.6
Niger	No	34.3	1.2	23.3	1.3	31.2	1.0
	Yes	11.4	1.1	23.7	0.6	7.4	1.4
Nigeria	No	34.8	6.6	24.3	6.1	31.3	6.7
	Yes	14.4	5.7	14.4	7.0	15.1	5.4
North Macedonia	No	37.2	12.7	35.5	14.2	19.5	32.6
	Yes	8.8	44.4	7.5	46.1	0.0	0.0
Pakistan	No	45.2	4.9	40.0	9.7	36.6	8.0
	Yes	36.5	10.2	35.2	6.7	43.9	10.8

Country	Child received prelacteal feed	Any prelacteal feeding		Milk-based only prelacteal feeding		Water-based only prelacteal feeding	
		Exclusive breastfeeding (%)	Formula consumption (%)	Exclusive breastfeeding (%)	Formula consumption (%)	Exclusive breastfeeding (%)	Formula consumption (%)
Panama	No	28.0	41.1	27.1	42.9	21.6	54.5
	Yes	8.5	81.5	8.9	80.7	8.0	79.3
Paraguay	No	33.1	20.9	34.0	21.6	30.8	28.2
	Yes	29.3	42.8	25.7	46.1	62.9	12.2
Peru	No	74.1	8.7	74.2	8.6	65.5	18.8
	Yes	53.4	33.4	52.9	33.8	96.6	3.4
Sao Tome and Principe	No	65.5	3.6	64.1	5.3	66.0	3.7
	Yes	54.6	19.7	85.3	14.7	48.2	21.5
Senegal	No	47.7	6.2	40.7	4.1	45.7	9.8
	Yes	35.9	7.2	44.4	26.0	33.1	1.5
Serbia	No	32.3	15.0	36.2	16.2	29.6	30.0
	Yes	23.7	39.4	14.5	49.6	0.0	39.1
Sierra Leone	No	54.4	7.7	52.9	8.6	54.4	8.2
	Yes	36.3	22.6	60.8	39.2	32.9	18.4
State of Palestine	No	47.5	26.3	46.6	27.1	40.8	34.3
	Yes	31.4	45.0	28.4	49.1	40.8	31.3
Sudan	No	59.8	3.3	56.2	3.0	58.7	3.5
	Yes	45.8	2.6	42.1	5.9	46.9	1.8
Suriname	No	13.1	51.3	12.3	52.0	9.8	56.5
	Yes	3.7	65.4	4.1	65.8	0.0	53.7
Tajikistan	No	36.6	9.2	34.6	11.1	36.4	10.2
	Yes	18.3	26.1	0.0	50.9	17.1	21.3
Tanzania	No	55.2	1.0	51.9	1.0	53.1	0.9
	Yes	40.2	1.0	29.2	0.0	42.9	1.2
Thailand	No	19.9	36.3	18.5	38.6	19.6	40.8
	Yes	13.7	64.9	19.4	64.9	1.6	65.8

Country	Child received prelacteal feed	Any prelacteal feeding		Milk-based only prelacteal feeding		Water-based only prelacteal feeding	
		Exclusive breastfeeding (%)	Formula consumption (%)	Exclusive breastfeeding (%)	Formula consumption (%)	Exclusive breastfeeding (%)	Formula consumption (%)
Togo	No	67.9	2.3	66.1	2.1	65.9	2.8
	Yes	50.0	5.6	35.9	33.9	59.7	1.6
Tonga	No	52.7	30.9	52.3	31.1	46.0	39.4
	Yes	14.2	78.7	10.8	84.1	63.9	0.0
Tunisia	No	17.9	44.1	16.9	47.0	14.5	48.6
	Yes	9.9	56.0	8.5	54.5	15.8	54.5
Turkey	No	34.4	18.2	33.9	19.9	30.5	31.3
	Yes	25.9	50.7	24.7	55.9	38.1	26.1
Ukraine	No	21.7	27.4	19.7	28.8	20.8	28.2
	Yes	0.4	48.6	1.8	68.5	0.0	50.2
Vietnam	No	34.6	17.8	22.4	29.9	26.0	35.9
	Yes	22.1	40.9	27.2	42.2	4.2	37.1
Yemen	No	16.0	21.1	10.8	30.7	11.2	32.9
	Yes	7.9	37.2	7.2	45.2	9.3	31.2
Zambia	No	73.1	1.5	72.8	1.5	72.6	1.5
	Yes	61.6	0.4	49.3	1.4	68.0	0.0
Zimbabwe	No	43.6	3.1	42.6	3.5	43.6	3.9
	Yes	33.8	8.1	27.1	19.2	16.4	0.0

*LMICs- low- and middle-income countries.

Table S4. Crude and adjusted association between types of prelacteal feedings with exclusive breastfeeding and formula consumption by regions of the world.

Type of prelacteal		Groups	Exclusive breastfeeding under six months						Formula consumption under six months						Number of countries
			Crude			Adjusted			Crude			Adjusted			
			PR	95% CI		PR	95% CI		PR	95% CI		PR	95% CI		
Any prelacteal feeding	All	All LMICs	0.60	0.56	0.64	0.62	0.59	0.66	1.89	1.72	2.08	1.72	1.59	1.85	85
	Regions of the world	West & Central Africa	0.53	0.44	0.63	0.54	0.46	0.64	1.50	1.24	1.83	1.49	1.28	1.73	22
		Eastern & Southern Africa	0.69	0.61	0.79	0.70	0.61	0.81	1.60	1.05	2.45	1.38	0.99	1.93	12
		Middle East & North Africa	0.61	0.51	0.74	0.63	0.53	0.75	1.65	1.50	1.81	1.57	1.45	1.71	7
		Eastern Europe & Central Asia	0.53	0.37	0.76	0.54	0.38	0.77	2.16	1.76	2.64	2.10	1.66	2.66	12
		South Asia	0.74	0.71	0.77	0.74	0.71	0.77	1.74	1.09	2.78	1.73	1.18	2.54	6
		East Asia & Pacific	0.64	0.55	0.74	0.69	0.59	0.81	2.60	2.20	3.06	2.32	1.98	2.71	9
		Latin America & Caribbean	0.54	0.47	0.62	0.59	0.51	0.67	2.04	1.66	2.52	1.69	1.48	1.94	17
	Income groups	Low income	0.60	0.52	0.70	0.61	0.53	0.70	1.62	1.32	1.98	1.45	1.22	1.72	23
		Lower-middle income	0.62	0.57	0.67	0.66	0.61	0.71	2.04	1.73	2.40	1.91	1.69	2.15	37
		Upper-middle income	0.55	0.48	0.63	0.55	0.48	0.64	1.83	1.61	2.07	1.62	1.47	1.79	25
Milk-only prelacteal feeding	All	All LMICs	0.69	0.64	0.73	0.73	0.68	0.78	2.38	2.12	2.68	1.81	1.67	1.97	79
	Regions of the world	West & Central Africa	0.91	0.78	1.06	0.96	0.82	1.11	3.36	2.22	5.10	1.96	1.55	2.47	19
		Eastern & Southern Africa	0.65	0.49	0.86	0.64	0.49	0.83	3.79	2.26	6.34	1.77	1.23	2.55	10
		Middle East & North Africa	0.67	0.56	0.79	0.71	0.58	0.87	1.66	1.44	1.93	1.56	1.38	1.76	7
		Eastern Europe & Central Asia	0.55	0.39	0.77	0.56	0.40	0.78	2.24	1.73	2.90	2.15	1.60	2.89	11
		South Asia	0.75	0.71	0.79	0.75	0.71	0.80	1.58	0.88	2.81	1.50	0.89	2.50	6
		East Asia & Pacific	0.71	0.59	0.86	0.78	0.63	0.96	2.67	2.06	3.47	2.21	1.70	2.87	9
		Latin America & Caribbean	0.56	0.49	0.64	0.66	0.60	0.73	2.19	1.76	2.72	1.72	1.49	1.98	17
	Income groups	Low income	0.84	0.71	0.99	0.85	0.71	1.01	3.92	2.81	5.46	2.02	1.62	2.51	19
		Lower-middle income	0.69	0.63	0.75	0.75	0.69	0.81	2.43	2.02	2.92	1.93	1.67	2.23	35
		Upper-middle income	0.60	0.52	0.69	0.61	0.53	0.71	1.95	1.69	2.25	1.64	1.47	1.84	25

Type of prelacteal	Groups		Exclusive breastfeeding under six months						Formula consumption under six months						Number of countries
			Crude			Adjusted			Crude			Adjusted			
			PR	95% CI		PR	95% CI		PR	95% CI		PR	95% CI		
Water-only prelacteal feeding	All	All LMICs	0.67	0.60	0.75	0.69	0.63	0.75	0.96	0.86	1.08	1.09	0.99	1.20	71
	Regions of the world	West & Central Africa	0.51	0.42	0.61	0.53	0.44	0.63	1.00	0.75	1.34	1.18	0.94	1.47	22
		Eastern & Southern Africa	0.71	0.56	0.89	0.72	0.58	0.91	0.80	0.58	1.10	1.09	0.73	1.63	10
		Middle East & North Africa	0.85	0.72	1.00	0.81	0.67	0.97	0.93	0.76	1.13	0.99	0.81	1.21	7
		Eastern Europe & Central Asia	0.75	0.47	1.19	0.77	0.44	1.33	1.24	0.82	1.88	1.13	0.74	1.71	6
		South Asia	0.91	0.80	1.04	0.92	0.81	1.04	1.10	0.90	1.36	1.09	0.89	1.34	4
		East Asia & Pacific	0.68	0.49	0.95	0.69	0.50	0.95	0.90	0.57	1.41	1.10	0.76	1.58	8
		Latin America & Caribbean	0.79	0.57	1.11	0.77	0.59	1.01	0.94	0.72	1.22	1.07	0.83	1.38	14
	Income groups	Low income	0.58	0.48	0.70	0.59	0.49	0.71	0.96	0.70	1.31	1.08	0.86	1.36	21
		Lower-middle income	0.73	0.65	0.82	0.74	0.67	0.82	0.98	0.85	1.12	1.11	0.98	1.26	32
		Upper-middle income	0.71	0.52	0.99	0.73	0.55	0.97	0.93	0.75	1.16	1.05	0.85	1.29	18

[*]LMICs- low- and middle-income countries; [**]PR – prevalence ratio; [***]Adjustments for: household wealth, area of residence, mother's level of education, mother's age, number of antenatal care visits, skilled birth attendant, institutional delivery, c-section delivery, sex of the child, and early initiation of breastfeeding.

Table S5. Crude and adjusted association between types of prelacteal feedings with exclusive breastfeeding by country.

Country	Exclusive breastfeeding under six months																	
	Any prelacteal feeding						Milk-based only prelacteal feeding						Water-based only prelacteal feeding					
	Crude			Adjusted***			Crude			Adjusted***			Crude			Adjusted***		
	PR**	95% CI		PR**	95% CI		PR**	95% CI		PR**	95% CI		PR**	95% CI		PR**	95% CI	
Afghanistan	0.83	0.72	0.95	0.83	0.72	0.96	0.77	0.41	1.45	0.81	0.42	1.57	0.88	0.76	1.02	0.88	0.75	1.02
Algeria	0.55	0.44	0.70	0.59	0.46	0.75	0.92	0.63	1.34	1.10	0.74	1.61	0.62	0.48	0.81	0.59	0.46	0.77
Angola	0.35	0.21	0.59	0.37	0.22	0.61	0.35	0.12	1.04	0.36	0.12	1.10	0.38	0.22	0.66	0.40	0.23	0.69
Armenia	0.67	0.23	1.92	0.63	0.24	1.66	0.59	0.13	2.63	0.61	0.16	2.39	0.81	0.25	2.61	0.68	0.21	2.16
Bangladesh	0.72	0.65	0.81	0.72	0.64	0.80	0.70	0.60	0.81	0.68	0.59	0.80	0.89	0.78	1.01	0.89	0.78	1.02
Belarus	0.25	0.12	0.53	0.28	0.14	0.56	0.41	0.18	0.90	0.44	0.21	0.93	*	*	*	*	*	*
Belize	0.44	0.16	1.18	0.44	0.18	1.07	0.28	0.07	1.22	0.32	0.07	1.44	0.32	0.04	2.48	0.31	0.04	2.66
Benin	0.44	0.33	0.60	0.47	0.35	0.63	0.34	0.11	1.07	0.35	0.11	1.08	0.49	0.36	0.66	0.52	0.38	0.71
Bhutan	0.77	0.47	1.27	0.77	0.48	1.26	0.68	0.37	1.26	0.68	0.37	1.22	*	*	*	*	*	*
Bolivia	0.51	0.37	0.70	0.55	0.41	0.75	0.53	0.38	0.75	0.59	0.42	0.82	0.62	0.30	1.29	0.69	0.37	1.30
Burkina Faso	0.14	0.09	0.23	0.14	0.09	0.24	*	*	*	*	*	*	0.14	0.08	0.23	0.14	0.09	0.24
Central African Republic	0.59	0.38	0.90	0.60	0.39	0.92	0.85	0.44	1.66	0.90	0.46	1.78	0.49	0.28	0.86	0.49	0.28	0.87
Cambodia	0.67	0.54	0.83	0.73	0.59	0.92	0.77	0.58	1.03	0.84	0.64	1.11	0.76	0.52	1.11	0.76	0.53	1.10
Cameroon	0.30	0.21	0.44	0.37	0.26	0.54	0.60	0.24	1.54	0.46	0.17	1.26	0.29	0.20	0.43	0.36	0.24	0.54
Chad	0.96	0.20	4.60	1.38	0.30	6.35	1.53	0.18	13.30	0.97	0.10	9.11	0.68	0.16	2.89	1.00	0.23	4.28
Colombia	0.61	0.51	0.74	0.61	0.50	0.73	0.68	0.56	0.84	0.67	0.55	0.82	0.62	0.43	0.90	0.63	0.43	0.91
Comoros	0.30	0.12	0.76	0.31	0.12	0.78	1.69	0.59	4.83	1.66	0.52	5.35	0.11	0.02	0.72	0.12	0.02	0.73
Congo Brazzaville	0.97	0.59	1.60	0.95	0.60	1.50	1.22	0.78	1.91	1.06	0.67	1.67	0.71	0.35	1.46	0.84	0.43	1.67
Congo Democratic Republic	0.85	0.64	1.14	0.83	0.64	1.08	1.25	0.79	2.00	1.19	0.72	1.96	0.70	0.48	1.03	0.72	0.49	1.04
Costa Rica	0.28	0.11	0.69	0.33	0.14	0.78	0.26	0.10	0.68	0.31	0.13	0.77	*	*	*	*	*	*
Cote d'Ivoire	0.36	0.25	0.52	0.37	0.26	0.54	0.71	0.22	2.24	0.93	0.31	2.84	0.35	0.24	0.53	0.35	0.24	0.53
Cuba	0.52	0.17	1.59	0.59	0.20	1.75	0.55	0.17	1.77	0.65	0.21	1.99	*	*	*	*	*	*
Dominican Republic	0.38	0.20	0.73	0.40	0.20	0.80	0.43	0.22	0.84	0.47	0.23	0.97	0.51	0.12	2.18	0.46	0.10	2.03
Egypt	0.85	0.73	0.99	0.86	0.74	1.00	0.83	0.58	1.19	0.85	0.59	1.22	0.96	0.82	1.12	0.97	0.83	1.13
El Salvador	0.55	0.36	0.84	0.62	0.40	0.96	0.55	0.36	0.86	0.64	0.40	1.02	0.59	0.20	1.74	0.55	0.22	1.39
Eswatini	0.63	0.43	0.93	0.62	0.42	0.91	*	*	*	*	*	*	0.68	0.46	0.99	0.67	0.46	0.98

Country	Exclusive breastfeeding under six months																	
	Any prelacteal feeding						Milk-based only prelacteal feeding						Water-based only prelacteal feeding					
	Crude			Adjusted***			Crude			Adjusted***			Crude			Adjusted***		
	PR**	95% CI		PR**	95% CI		PR**	95% CI		PR**	95% CI		PR**	95% CI		PR**	95% CI	
Ethiopia	0.91	0.74	1.12	0.93	0.74	1.17	0.20	0.07	0.53	0.19	0.07	0.53	1.20	1.01	1.43	1.26	1.04	1.53
Gabon	0.38	0.11	1.31	0.36	0.12	1.09	0.33	0.05	2.41	0.28	0.05	1.52	0.77	0.16	3.64	0.91	0.21	3.89
Gambia	0.64	0.44	0.92	0.65	0.45	0.93	0.75	0.20	2.84	0.81	0.21	3.22	0.58	0.39	0.86	0.59	0.39	0.87
Ghana	0.33	0.20	0.55	0.35	0.21	0.58	0.63	0.28	1.42	0.71	0.31	1.65	0.27	0.12	0.60	0.28	0.13	0.63
Guatemala	0.47	0.39	0.57	0.59	0.49	0.72	0.46	0.37	0.58	0.63	0.51	0.79	0.77	0.58	1.01	0.71	0.56	0.91
Guinea	0.58	0.43	0.77	0.59	0.44	0.79	0.76	0.21	2.72	0.64	0.17	2.38	0.58	0.43	0.77	0.60	0.44	0.81
Guinea Bissau	0.86	0.68	1.09	0.85	0.66	1.08	*	*	*	*	*	*	0.88	0.69	1.12	0.86	0.67	1.10
Guyana	0.53	0.28	1.00	0.50	0.25	0.98	0.37	0.15	0.94	0.38	0.15	0.97	1.15	0.49	2.68	1.02	0.45	2.31
Haiti	0.41	0.27	0.62	0.43	0.29	0.64	0.24	0.04	1.58	0.28	0.04	1.88	0.45	0.30	0.68	0.47	0.31	0.69
Honduras	0.54	0.43	0.70	0.62	0.49	0.79	0.56	0.40	0.78	0.70	0.51	0.95	0.99	0.73	1.34	0.93	0.69	1.25
India	0.73	0.70	0.77	0.73	0.70	0.77	0.75	0.71	0.80	0.76	0.71	0.81	0.81	0.74	0.88	0.83	0.76	0.90
Indonesia	0.67	0.59	0.77	0.68	0.59	0.78	0.64	0.56	0.74	0.66	0.57	0.77	1.06	0.84	1.35	1.03	0.82	1.31
Iraq	0.53	0.42	0.66	0.54	0.43	0.68	0.51	0.38	0.70	0.53	0.38	0.74	0.96	0.73	1.28	0.92	0.68	1.23
Jamaica	0.73	0.30	1.76	0.75	0.33	1.72	0.76	0.29	1.96	0.78	0.33	1.86	1.11	0.25	4.88	1.38	0.29	6.44
Jordan	0.60	0.44	0.82	0.65	0.48	0.88	0.65	0.46	0.94	0.73	0.52	1.04	0.62	0.35	1.11	0.59	0.33	1.06
Kazakhstan	0.34	0.18	0.64	0.40	0.22	0.74	0.45	0.23	0.88	0.53	0.27	1.05	0.18	0.04	0.71	0.20	0.05	0.76
Kiribati	0.84	0.65	1.08	0.84	0.66	1.09	0.66	0.34	1.28	0.77	0.39	1.52	0.93	0.72	1.20	0.88	0.68	1.13
Kosovo	0.57	0.30	1.05	0.59	0.31	1.12	0.52	0.27	1.00	0.51	0.26	1.01	*	*	*	*	*	*
Kyrgyzstan	0.85	0.54	1.34	0.86	0.55	1.37	0.90	0.52	1.54	0.92	0.53	1.58	0.89	0.42	1.91	0.93	0.45	1.93
Lao	0.41	0.30	0.54	0.45	0.34	0.60	0.54	0.38	0.77	0.63	0.45	0.90	0.42	0.26	0.68	0.44	0.27	0.70
Lesotho	0.89	0.57	1.38	0.93	0.62	1.39	0.53	0.20	1.43	0.62	0.24	1.63	0.84	0.41	1.71	0.78	0.41	1.50
Liberia	0.36	0.18	0.74	0.37	0.19	0.70	*	*	*	*	*	*	0.35	0.17	0.74	0.36	0.18	0.70
Madagascar	0.61	0.52	0.72	0.62	0.53	0.73	0.88	0.57	1.38	0.77	0.48	1.25	0.61	0.51	0.71	0.63	0.53	0.75
Mali	0.58	0.45	0.74	0.59	0.46	0.75	0.77	0.46	1.29	0.78	0.47	1.30	0.59	0.45	0.78	0.60	0.45	0.80
Mauritania	0.56	0.43	0.73	0.61	0.47	0.78	0.69	0.46	1.03	0.78	0.54	1.12	0.59	0.42	0.83	0.60	0.43	0.83
Mexico	0.48	0.29	0.80	0.54	0.35	0.82	0.52	0.31	0.87	0.60	0.39	0.92	0.22	0.05	0.95	0.19	0.04	0.94
Moldova	0.59	0.31	1.10	0.63	0.34	1.15	0.36	0.15	0.88	0.42	0.17	1.03	1.05	0.44	2.48	1.01	0.37	2.70
Mongolia	0.68	0.48	0.95	0.77	0.56	1.07	0.54	0.33	0.87	0.62	0.40	0.97	1.02	0.62	1.67	1.09	0.73	1.62

Country	Exclusive breastfeeding under six months																	
	Any prelacteal feeding						Milk-based only prelacteal feeding						Water-based only prelacteal feeding					
	Crude			Adjusted***			Crude			Adjusted***			Crude			Adjusted***		
	PR**	95% CI		PR**	95% CI		PR**	95% CI		PR**	95% CI		PR**	95% CI		PR**	95% CI	
Montenegro	1.73	0.81	3.72	2.01	0.87	4.64	1.53	0.70	3.32	1.75	0.75	4.09	*	*	*	*	*	*
Mozambique	0.60	0.36	0.98	0.59	0.36	0.96	0.79	0.40	1.57	0.73	0.35	1.49	0.50	0.26	0.97	0.51	0.27	0.99
Myanmar	0.69	0.50	0.95	0.79	0.58	1.08	0.83	0.60	1.15	0.94	0.68	1.30	0.32	0.11	0.92	0.40	0.15	1.04
Namibia	0.49	0.31	0.79	0.49	0.31	0.79	0.40	0.19	0.85	0.39	0.18	0.82	0.75	0.39	1.44	0.81	0.40	1.61
Nepal	0.76	0.56	1.03	0.77	0.55	1.06	0.73	0.53	1.02	0.74	0.52	1.06	*	*	*	*	*	*
Niger	0.33	0.25	0.45	0.32	0.24	0.43	1.02	0.72	1.45	1.27	0.87	1.83	0.24	0.15	0.38	0.23	0.14	0.36
Nigeria	0.41	0.33	0.52	0.46	0.37	0.57	0.59	0.33	1.06	0.74	0.40	1.34	0.48	0.38	0.62	0.53	0.42	0.68
North Macedonia	0.24	0.08	0.68	0.15	0.03	0.67	0.21	0.07	0.64	0.19	0.06	0.58	*	*	*	*	*	*
Pakistan	0.81	0.66	0.99	0.79	0.66	0.96	0.88	0.67	1.15	0.86	0.66	1.12	1.20	0.96	1.49	1.22	0.98	1.52
Panama	0.30	0.11	0.80	0.29	0.12	0.71	0.33	0.12	0.87	0.31	0.12	0.80	0.37	0.04	3.78	0.39	0.06	2.70
Paraguay	0.88	0.55	1.42	0.85	0.52	1.38	0.76	0.45	1.27	0.75	0.44	1.29	2.04	1.13	3.70	1.77	0.88	3.56
Peru	0.72	0.65	0.80	0.79	0.70	0.90	0.71	0.64	0.80	0.79	0.70	0.89	1.48	1.35	1.61	1.31	1.08	1.60
Sao Tome and Principe	0.83	0.54	1.28	0.80	0.52	1.23	1.33	0.90	1.96	1.37	1.01	1.85	0.73	0.43	1.25	0.70	0.42	1.19
Senegal	0.75	0.57	0.99	0.71	0.54	0.92	1.09	0.71	1.67	1.06	0.68	1.65	0.72	0.52	1.00	0.70	0.52	0.94
Serbia	0.73	0.24	2.21	0.67	0.27	1.65	0.40	0.12	1.36	0.44	0.15	1.29	*	*	*	*	*	*
Sierra Leone	0.67	0.46	0.96	0.68	0.48	0.99	1.15	0.40	3.30	1.38	0.50	3.77	0.61	0.42	0.86	0.61	0.42	0.87
State of Palestine	0.66	0.52	0.85	0.67	0.51	0.87	0.61	0.46	0.81	0.63	0.47	0.84	1.00	0.64	1.57	0.87	0.56	1.37
Sudan	0.77	0.65	0.90	0.76	0.65	0.89	0.75	0.51	1.11	0.75	0.50	1.12	0.80	0.68	0.94	0.80	0.68	0.94
Suriname	0.28	0.10	0.80	0.28	0.09	0.86	0.34	0.12	0.97	0.36	0.11	1.13	*	*	*	*	*	*
Tajikistan	0.50	0.26	0.96	0.51	0.26	1.00	*	*	*	*	*	*	0.47	0.22	0.98	0.47	0.22	1.01
Tanzania	0.73	0.58	0.92	0.71	0.57	0.88	*	*	*	*	*	*	0.81	0.63	1.03	0.81	0.64	1.02
Thailand	0.69	0.34	1.41	0.47	0.22	1.00	1.05	0.48	2.29	0.63	0.26	1.52	0.08	0.01	0.47	0.09	0.02	0.52
Togo	0.74	0.55	0.99	0.77	0.57	1.03	0.54	0.22	1.31	0.57	0.23	1.41	0.91	0.63	1.29	0.95	0.67	1.34
Tonga	0.27	0.09	0.77	0.32	0.11	0.94	0.21	0.06	0.71	0.25	0.07	0.91	*	*	*	*	*	*
Tunisia	0.55	0.28	1.11	0.52	0.26	1.04	0.50	0.21	1.20	0.46	0.20	1.09	1.09	0.35	3.37	1.12	0.35	3.59
Turkey	0.75	0.48	1.18	0.67	0.43	1.03	0.73	0.45	1.17	0.61	0.38	0.97	1.25	0.66	2.36	1.64	0.94	2.87
Vietnam	0.02	0.00	0.14	0.02	0.00	0.13	0.09	0.01	0.76	0.08	0.01	0.68	0.16	0.02	1.16	0.12	0.02	0.76
Ukraine	0.64	0.42	0.98	0.95	0.56	1.63	1.21	0.78	1.87	1.72	1.05	2.82	*	*	*	*	*	*

Country	Exclusive breastfeeding under six months																	
	Any prelacteal feeding						Milk-based only prelacteal feeding						Water-based only prelacteal feeding					
	Crude			Adjusted***			Crude			Adjusted***			Crude			Adjusted***		
	PR**	95% CI		PR**	95% CI		PR**	95% CI		PR**	95% CI		PR**	95% CI		PR**	95% CI	
Yemen	0.49	0.33	0.73	0.47	0.31	0.73	0.67	0.30	1.50	0.68	0.31	1.51	0.83	0.57	1.21	0.81	0.55	1.18
Zambia	0.84	0.67	1.07	0.86	0.69	1.09	0.68	0.38	1.21	0.69	0.38	1.26	*	*	*	*	*	*
Zimbabwe	0.78	0.55	1.10	0.89	0.62	1.27	0.64	0.22	1.83	0.77	0.27	2.20	*	*	*	*	*	*

*Due to the small number of children (<3) it was not possible to run the analysis; **PR – prevalence ratio; ***Adjustments for: household wealth, area of residence, mother's level of education, mother's age, number of antenatal care visits, skilled birth attendant, institutional delivery, c-section delivery, sex of the child, and early initiation of breastfeeding.

Table S6. Crude and adjusted association between types of prelacteal feedings and formula consumption by country.

Country	Formula consumption under six months																	
	Any prelacteal feeding						Milk-based only prelacteal feeding						Water-based only prelacteal feeding					
	Crude			Adjusted			Crude			Adjusted			Crude			Adjusted		
	PR**	95% CI		PR**	95% CI		PR**	95% CI		PR**	95% CI		PR**	95% CI		PR**	95% CI	
Afghanistan	1.49	1.03	2.15	1.56	1.08	2.24	1.90	0.87	4.16	1.88	0.80	4.43	0.98	0.64	1.49	1.02	0.69	1.53
Algeria	2.01	1.56	2.57	1.80	1.38	2.34	1.57	1.22	2.03	1.29	0.97	1.72	1.26	0.99	1.59	1.37	1.09	1.71
Angola	4.48	2.70	7.41	2.11	1.21	3.69	9.74	6.00	15.81	2.49	1.18	5.26	0.59	0.20	1.72	0.83	0.30	2.27
Armenia	0.72	0.20	2.53	0.65	0.18	2.38	0.75	0.14	3.90	0.58	0.12	2.87	0.72	0.10	4.96	0.84	0.12	5.76
Bangladesh	2.63	2.04	3.38	2.50	1.94	3.23	2.87	2.20	3.74	2.64	2.00	3.48	1.13	0.77	1.66	1.14	0.77	1.69
Belarus	1.50	1.00	2.23	1.46	0.95	2.22	1.23	0.83	1.84	1.21	0.82	1.81	*	*	*	*	*	*
Belize	1.77	1.22	2.58	1.96	1.26	3.05	2.00	1.35	2.97	1.90	1.09	3.31	0.99	0.32	3.08	1.58	0.55	4.58
Benin	1.93	0.76	4.90	2.09	0.75	5.84	3.05	0.64	14.51	2.62	0.62	11.09	1.81	0.68	4.81	2.01	0.69	5.84
Bhutan	0.60	0.14	2.49	0.62	0.11	3.47	1.12	0.27	4.61	1.00	0.20	5.06	*	*	*	*	*	*
Bolivia	3.32	2.26	4.87	2.68	1.76	4.09	3.24	2.28	4.63	2.69	1.79	4.03	1.14	0.45	2.93	0.88	0.35	2.20
Burkina Faso	1.74	0.40	7.55	2.75	0.62	12.25	*	*	*	*	*	*	0.44	0.08	2.46	0.96	0.13	7.09
Central African Republic	2.46	1.02	5.93	1.22	0.63	2.37	9.49	4.12	21.87	2.42	1.27	4.62	0.88	0.13	6.00	0.75	0.12	4.52
Cambodia	3.66	2.17	6.18	2.78	1.65	4.69	3.56	2.01	6.31	2.43	1.37	4.32	0.58	0.22	1.53	0.88	0.36	2.13
Cameroon	1.01	0.57	1.79	2.30	1.43	3.69	6.39	3.35	12.16	3.44	1.39	8.50	0.75	0.42	1.36	1.77	1.07	2.92
Chad	0.84	0.35	2.03	1.29	0.64	2.58	3.96	1.80	8.73	2.36	1.29	4.30	0.33	0.19	0.55	0.60	0.36	0.98
Colombia	1.66	1.38	1.99	1.56	1.29	1.88	1.79	1.49	2.14	1.65	1.36	2.00	0.64	0.42	0.98	0.73	0.48	1.11
Comoros	0.92	0.55	1.53	0.80	0.51	1.26	1.43	0.63	3.26	0.92	0.50	1.69	0.73	0.43	1.24	0.76	0.46	1.27
Congo Brazzaville	1.34	0.66	2.70	1.24	0.61	2.53	2.59	1.28	5.23	2.09	1.04	4.23	0.44	0.11	1.67	0.59	0.14	2.43
Congo Democratic Republic	2.87	1.28	6.47	1.53	0.79	2.95	4.16	1.10	15.79	1.93	0.53	7.11	2.90	1.11	7.63	1.70	0.77	3.78
Costa Rica	2.08	1.21	3.59	1.94	1.20	3.13	2.09	1.21	3.60	1.93	1.19	3.13	*	*	*	*	*	*
Cote d'Ivoire	2.14	0.75	6.09	1.96	0.80	4.80	9.99	4.44	22.50	2.39	0.39	14.67	0.96	0.45	2.06	1.67	0.66	4.23
Cuba	1.74	0.30	10.22	0.83	0.32	2.16	1.96	0.34	11.42	1.14	0.50	2.62	*	*	*	*	*	*
Dominican Republic	1.42	1.03	1.96	1.31	0.95	1.80	1.31	0.98	1.74	1.15	0.86	1.54	1.43	0.91	2.23	1.96	1.29	2.99
Egypt	1.81	1.26	2.60	1.73	1.19	2.52	2.50	1.65	3.78	2.25	1.47	3.43	0.92	0.67	1.26	0.89	0.64	1.23
El Salvador	2.62	1.85	3.70	2.09	1.40	3.12	2.57	1.80	3.68	2.02	1.31	3.09	1.56	0.66	3.73	1.75	0.94	3.25
Eswatini	0.91	0.24	3.46	1.43	0.26	7.86	*	*	*	*	*	*	0.98	0.26	3.71	1.46	0.27	8.02

Country	Formula consumption under six months																	
	Any prelacteal feeding						Milk-based only prelacteal feeding						Water-based only prelacteal feeding					
	Crude			Adjusted			Crude			Adjusted			Crude			Adjusted		
	PR**	95% CI		PR**	95% CI		PR**	95% CI		PR**	95% CI		PR**	95% CI		PR**	95% CI	
Ethiopia	0.90	0.27	2.99	0.96	0.31	2.95	1.90	0.33	10.96	1.68	0.55	5.13	0.68	0.18	2.60	0.83	0.20	3.42
Gabon	1.37	1.14	1.64	1.27	1.05	1.52	1.47	1.25	1.73	1.33	1.08	1.64	0.95	0.73	1.24	0.96	0.74	1.23
Gambia	1.90	0.75	4.79	1.87	0.77	4.49	5.33	0.91	31.35	3.39	0.37	31.01	1.73	0.64	4.68	1.68	0.67	4.23
Ghana	1.96	1.21	3.20	1.61	1.04	2.50	1.44	0.64	3.25	0.94	0.37	2.42	1.62	0.92	2.86	1.43	0.86	2.37
Guatemala	5.73	4.22	7.78	2.97	2.14	4.12	5.75	4.38	7.54	2.69	1.93	3.76	0.58	0.23	1.45	0.72	0.36	1.43
Guinea	1.08	0.52	2.26	1.27	0.64	2.51	2.45	0.62	9.77	0.96	0.24	3.87	0.91	0.43	1.96	1.21	0.61	2.43
Guinea Bissau	3.59	1.21	10.66	3.98	1.55	10.19	*	*	*	*	*	*	2.61	0.78	8.74	2.80	0.99	7.91
Guyana	1.53	1.04	2.26	1.50	1.02	2.20	1.84	1.27	2.65	1.75	1.17	2.62	0.35	0.09	1.42	0.40	0.12	1.29
Haiti	1.61	1.06	2.46	1.42	0.97	2.07	3.64	1.91	6.93	2.35	1.27	4.34	1.24	0.74	2.06	1.16	0.75	1.80
Honduras	2.24	1.72	2.92	1.73	1.32	2.28	2.51	1.98	3.17	1.77	1.38	2.26	0.66	0.41	1.07	0.80	0.51	1.25
India	1.03	0.80	1.32	1.15	0.87	1.51	1.07	0.78	1.46	1.17	0.84	1.63	1.03	0.69	1.54	1.13	0.75	1.70
Indonesia	2.57	2.07	3.20	2.59	2.06	3.24	2.68	2.17	3.31	2.51	2.01	3.13	0.36	0.20	0.63	0.45	0.25	0.81
Iraq	1.64	1.38	1.94	1.50	1.26	1.79	1.99	1.72	2.30	1.79	1.53	2.09	0.60	0.46	0.77	0.66	0.50	0.85
Jamaica	1.03	0.67	1.59	0.86	0.55	1.33	1.02	0.64	1.64	0.85	0.54	1.33	1.44	0.80	2.57	1.71	0.71	4.08
Jordan	1.52	1.29	1.81	1.52	1.28	1.82	1.59	1.36	1.85	1.55	1.32	1.83	0.91	0.61	1.37	0.99	0.66	1.49
Kazakhstan	2.55	1.55	4.19	1.98	1.12	3.51	2.70	1.59	4.57	2.30	1.24	4.23	1.25	0.36	4.32	0.93	0.36	2.35
Kiribati	2.19	1.00	4.82	3.28	1.42	7.56	4.34	1.83	10.29	4.13	1.92	8.91	1.01	0.39	2.66	1.43	0.60	3.43
Kosovo	2.74	1.71	4.41	2.52	1.52	4.19	2.87	1.79	4.58	2.59	1.57	4.26	*	*	*	*	*	*
Kyrgyzstan	2.29	1.09	4.82	1.98	0.85	4.62	2.18	0.88	5.39	1.96	0.70	5.46	0.97	0.25	3.74	0.74	0.20	2.82
Lao	3.28	2.21	4.86	2.43	1.66	3.55	3.75	2.56	5.48	2.15	1.42	3.25	1.00	0.52	1.93	1.27	0.69	2.36
Lesotho	2.74	1.32	5.70	2.06	1.04	4.10	4.11	1.98	8.54	2.38	0.91	6.21	2.26	0.86	5.97	2.80	1.28	6.09
Liberia	0.60	0.17	2.18	0.57	0.15	2.11	*	*	*	*	*	*	0.62	0.17	2.23	0.57	0.15	2.11
Madagascar	2.11	0.86	5.16	2.65	1.16	6.03	9.90	3.78	25.91	3.29	1.15	9.41	0.76	0.27	2.15	1.65	0.62	4.41
Mali	0.80	0.34	1.93	0.77	0.32	1.89	1.71	0.50	5.84	1.89	0.60	5.97	0.61	0.20	1.85	0.65	0.21	1.96
Mauritania	1.55	0.91	2.65	1.30	0.80	2.12	2.21	1.13	4.31	1.49	0.80	2.75	1.06	0.57	1.97	1.22	0.67	2.19
Mexico	1.80	1.36	2.38	1.62	1.25	2.09	1.83	1.39	2.41	1.62	1.26	2.08	0.51	0.17	1.54	0.57	0.20	1.62
Moldova	2.27	1.29	3.97	2.56	1.39	4.72	3.13	1.80	5.43	3.15	1.76	5.64	0.50	0.07	3.33	0.55	0.10	3.16
Mongolia	2.24	1.33	3.76	1.50	0.89	2.53	2.50	1.42	4.40	1.64	0.95	2.85	1.04	0.31	3.50	0.89	0.37	2.15

Country	Formula consumption under six months																	
	Any prelacteal feeding						Milk-based only prelacteal feeding						Water-based only prelacteal feeding					
	Crude			Adjusted			Crude			Adjusted			Crude			Adjusted		
	PR**	95% CI		PR**	95% CI		PR**	95% CI		PR**	95% CI		PR**	95% CI		PR**	95% CI	
Montenegro	1.09	0.55	2.17	0.94	0.48	1.84	1.14	0.57	2.27	0.95	0.48	1.88	*	*	*	*	*	*
Mozambique	2.47	0.95	6.37	1.50	0.52	4.30	4.95	1.73	14.17	1.62	0.53	4.96	1.71	0.42	6.89	1.36	0.46	4.03
Myanmar	5.36	2.19	13.15	5.04	1.86	13.65	5.85	2.42	14.14	5.10	1.78	14.64	1.36	0.19	9.60	1.21	0.19	7.64
Namibia	1.62	0.88	2.99	1.64	0.87	3.09	2.79	1.49	5.21	2.55	1.27	5.10	0.31	0.07	1.36	0.46	0.10	2.05
Nepal	4.43	1.63	12.03	3.47	1.06	11.32	3.53	1.24	10.08	2.46	0.76	7.98	*	*	*	*	*	*
Niger	0.96	0.37	2.55	1.37	0.56	3.37	0.45	0.05	3.65	1.05	0.12	9.32	1.36	0.48	3.89	1.40	0.51	3.87
Nigeria	0.85	0.59	1.23	1.24	0.80	1.91	1.15	0.51	2.62	1.86	0.82	4.21	0.80	0.55	1.16	1.05	0.68	1.61
North Macedonia	3.49	1.35	9.02	4.09	1.77	9.45	3.24	1.39	7.57	3.58	1.72	7.47	*	*	*	*	*	*
Pakistan	2.09	1.15	3.81	1.96	1.08	3.54	0.69	0.37	1.28	0.73	0.42	1.29	1.35	0.85	2.14	1.08	0.71	1.64
Panama	1.98	1.51	2.61	1.59	1.21	2.11	1.88	1.44	2.46	1.54	1.16	2.06	1.46	0.86	2.46	1.43	1.03	1.99
Paraguay	2.05	1.39	3.04	1.88	1.24	2.84	2.14	1.46	3.13	1.81	1.20	2.72	0.43	0.10	1.96	1.01	0.32	3.23
Peru	3.84	2.86	5.15	2.50	1.78	3.53	3.91	2.92	5.24	2.54	1.80	3.58	0.18	0.02	1.42	0.24	0.03	1.87
Sao Tome and Principe	5.45	1.73	17.16	3.91	1.52	10.08	2.80	0.30	26.50	10.43	1.92	56.75	5.77	1.79	18.62	3.68	1.28	10.54
Senegal	1.17	0.50	2.73	0.88	0.42	1.85	6.35	2.74	14.76	2.01	0.96	4.21	0.15	0.05	0.45	0.27	0.09	0.77
Serbia	2.63	1.07	6.46	2.37	1.07	5.24	3.06	1.13	8.25	3.50	1.66	7.38	*	*	*	*	*	*
Sierra Leone	2.93	1.55	5.55	2.36	1.34	4.16	4.57	0.86	24.16	3.06	0.86	10.87	2.26	1.09	4.65	1.83	0.95	3.54
State of Palestine	1.71	1.34	2.18	1.62	1.25	2.09	1.81	1.44	2.28	1.68	1.31	2.17	0.91	0.51	1.62	1.11	0.61	2.03
Sudan	0.78	0.36	1.72	0.64	0.27	1.48	1.95	0.51	7.47	1.37	0.32	5.92	0.52	0.21	1.26	0.46	0.18	1.13
Suriname	1.27	0.98	1.66	1.27	0.98	1.66	1.27	0.97	1.65	1.24	0.96	1.61	*	*	*	*	*	*
Tajikistan	2.83	1.60	4.99	2.80	1.49	5.24	*	*	*	*	*	*	2.09	1.08	4.03	2.01	0.97	4.17
Tanzania	1.00	0.11	8.79	1.78	0.65	4.92	*	*	*	*	*	*	1.39	0.16	12.21	2.01	0.68	5.92
Thailand	1.79	1.21	2.64	2.04	1.44	2.89	1.68	1.11	2.55	2.14	1.39	3.28	1.61	1.11	2.34	1.64	1.15	2.34
Togo	2.41	0.74	7.84	1.09	0.30	3.95	15.79	5.05	49.34	8.62	2.24	33.21	0.55	0.07	4.47	0.16	0.02	1.30
Tonga	2.55	1.63	3.99	2.13	1.30	3.48	2.70	1.77	4.14	2.22	1.29	3.82	*	*	*	*	*	*
Tunisia	1.27	0.97	1.65	1.38	1.06	1.79	1.16	0.88	1.53	1.25	0.95	1.64	1.12	0.71	1.78	1.15	0.74	1.78
Turkey	2.79	1.82	4.28	2.98	1.91	4.64	2.81	1.87	4.21	2.86	1.84	4.44	0.83	0.39	1.80	0.91	0.41	2.05
Vietnam	2.30	1.25	4.23	1.64	0.92	2.92	2.38	1.35	4.17	2.33	1.14	4.79	1.03	0.53	2.02	1.42	0.74	2.73
Ukraine	1.77	0.96	3.29	2.14	1.16	3.93	1.41	0.99	2.02	1.17	0.82	1.66	*	*	*	*	*	*

Country	Formula consumption under six months																	
	Any prelacteal feeding						Milk-based only prelacteal feeding						Water-based only prelacteal feeding					
	Crude			Adjusted			Crude			Adjusted			Crude			Adjusted		
	PR**	95% CI		PR**	95% CI		PR**	95% CI		PR**	95% CI		PR**	95% CI		PR**	95% CI	
Yemen	1.77	1.43	2.18	1.69	1.36	2.10	1.47	1.14	1.90	1.41	1.08	1.82	0.95	0.80	1.13	0.98	0.83	1.15
Zambia	0.28	0.04	2.20	0.13	0.01	1.17	0.97	0.12	7.80	0.17	0.02	1.77	*	*	*	*	*	*
Zimbabwe	2.60	0.95	7.10	1.76	0.62	5.00	5.56	1.46	21.14	1.87	0.29	12.27	*	*	*	*	*	*

*Due to the small number of children (<3) it was not possible to run the analysis; **PR – prevalence ratio; ***Adjustments for: household wealth, area of residence, mother's level of education, mother's age, number of antenatal care visits, skilled birth attendant, institutional delivery, c-section delivery, sex of the child, and early initiation of breastfeeding.

Table S7. Food types** consumed by non-exclusively breastfed children in the day previous the survey by country.

Country	Year	Any breastfeeding (%)		Plain water (%)		Other liquids (%)		Other milks (%)		Complementary foods (%)	
		Milk-based only prelacteals	Water-based only prelacteals	Milk-based only prelacteals	Water-based only prelacteals	Milk-based only prelacteals	Water-based only prelacteals	Milk-based only prelacteals	Water-based only prelacteals	Milk-based only prelacteals	Water-based only prelacteals
Afghanistan	2015	97.8	98.1	29.1	32.8	9.5	12.6	54.7	42.2	16.6	13.9
Algeria	2018	83.0	87.9	48.1	64.4	13.9	22.2	47.3	37.9	21.6	21.4
Angola	2015	90.5	99.9	60.6	75.5	15.7	22.2	60.7	5.8	20.5	37.1
Armenia	2010	44.0	84.8	73.4	71.7	57.3	50.8	67.9	26.6	0.0	9.0
Bangladesh	2019	97.7	97.7	31.1	29.0	3.3	2.5	41.7	21.8	15.1	14.5
Belarus	2012	76.7	73.0	62.3	73.0	28.1	30.3	59.1	74.8	29.1	12.2
Belize	2015	92.8	100.0	45.6	80.8	42.5	61.6	90.4	41.6	31.2	43.8
Benin	2014	98.2	98.8	70.3	70.6	12.1	18.9	14.9	7.3	21.9	30.4
Bhutan	2010	100.0	100.0	49.7	32.1	12.7	0.0	11.9	16.9	54.6	35.7
Bolivia	2016	94.9	100.0	18.7	30.1	8.7	22.1	56.4	43.6	6.8	0.0
Burkina Faso	2010	100.0	99.7	84.0	94.4	11.3	11.4	11.3	2.4	0.0	4.8
Central African Republic	2018	97.2	94.8	54.3	67.0	17.6	27.8	24.4	6.0	51.0	50.8
Cambodia	2014	88.1	97.4	31.0	35.8	1.5	6.3	33.3	12.2	10.3	10.2
Cameroon	2014	100.0	98.1	28.2	83.8	65.2	10.8	63.2	9.5	11.7	19.3
Chad	2014	94.1	98.7	99.6	99.5	32.8	12.1	24.7	10.6	22.2	16.1
Colombia	2010	92.7	95.0	40.8	57.9	13.7	22.8	54.6	37.8	15.9	34.9
Comoros	2012	90.8	99.5	80.0	97.4	8.2	33.9	36.6	30.1	14.5	38.7
Congo Brazzaville	2014	99.3	99.5	46.4	73.1	20.7	18.0	29.8	12.4	25.8	28.8
Congo Democratic Republic	2017	100.0	98.5	8.9	53.7	6.5	15.9	21.3	15.5	15.8	31.2
Costa Rica	2018	89.4	52.6	51.1	100.0	26.5	47.4	62.0	47.4	27.9	100.0
Cote d'Ivoire	2016	100.0	99.7	76.1	87.8	19.2	11.0	51.5	7.2	7.6	19.0
Cuba	2019	75.8	100.0	75.0	79.0	37.1	0.0	28.6	79.0	13.5	79.0
Dominican Republic	2014	77.1	92.2	72.4	77.2	27.1	25.8	89.7	90.1	16.0	16.0
Egypt	2014	93.5	98.5	39.6	42.4	18.8	21.7	38.0	17.5	9.8	14.9

Country	Year	Any breastfeeding (%)		Plain water (%)		Other liquids (%)		Other milks (%)		Complementary foods (%)	
		Milk-based only prelacteals	Water-based only prelacteals	Milk-based only prelacteals	Water-based only prelacteals	Milk-based only prelacteals	Water-based only prelacteals	Milk-based only prelacteals	Water-based only prelacteals	Milk-based only prelacteals	Water-based only prelacteals
El Salvador	2014	91.5	59.4	35.8	56.8	16.2	11.3	58.6	47.1	14.6	11.3
Eswatini	2014	*	96.1	*	22.8	*	3.9	*	9.9	*	39.1
Ethiopia	2011	100.0	99.7	29.1	34.7	6.3	3.9	51.2	10.2	22.8	3.4
Gabon	2012	93.3	88.3	73.5	91.0	8.5	5.9	87.0	61.4	13.8	27.7
Gambia	2018	100.0	97.7	42.1	60.0	16.3	10.3	33.8	9.5	7.6	10.2
Ghana	2017	100.0	97.8	72.7	87.2	11.2	4.8	25.0	27.9	30.8	28.3
Guatemala	2014	94.3	100.0	38.1	42.6	12.0	20.4	55.0	13.0	10.7	10.8
Guinea	2016	100.0	99.0	64.7	68.8	0.0	13.0	22.3	11.0	29.0	31.1
Guinea Bissau	2018	100.0	99.4	100.0	40.3	100.0	15.7	0.0	5.9	100.0	12.0
Guyana	2014	71.8	100.0	76.9	51.8	25.2	36.3	81.2	44.2	25.6	7.8
Haiti	2012	92.7	99.4	90.3	77.6	35.6	27.3	60.3	23.7	77.5	54.0
Honduras	2011	91.3	95.6	37.0	38.1	23.4	24.8	66.0	37.2	18.7	17.6
India	2015	97.3	98.2	43.7	47.5	7.4	10.3	29.2	22.0	9.1	13.0
Indonesia	2017	87.4	96.1	29.8	36.3	2.1	5.8	44.7	9.9	16.8	21.1
Iraq	2018	69.1	83.8	52.5	58.7	18.6	16.3	70.2	28.9	21.6	21.4
Jamaica	2011	95.4	73.8	59.1	72.4	27.2	46.2	51.7	72.4	2.8	46.2
Jordan	2017	83.3	86.6	51.0	60.9	15.8	25.1	67.8	50.8	23.1	37.4
Kazakhstan	2015	73.7	100.0	78.0	93.0	17.4	6.5	54.9	23.7	19.0	14.4
Kiribati	2018	78.7	98.6	0.0	28.1	10.7	10.4	42.7	13.3	0.0	3.6
Kosovo	2019	73.4	100.0	47.0	42.2	22.5	42.2	65.0	0.0	21.1	42.2
Kyrgyzstan	2018	100.0	97.9	38.1	58.0	26.3	2.3	25.4	12.3	44.4	16.0
Lao	2017	81.3	89.8	45.7	68.7	11.6	7.8	42.1	15.3	28.6	50.5
Lesotho	2018	56.4	90.7	34.4	9.3	25.2	20.6	60.1	36.7	25.2	15.9
Liberia	2013	100.0	99.5	0.0	77.7	0.0	0.5	0.0	4.5	0.0	27.7
Madagascar	2018	94.3	99.7	32.4	50.2	22.6	29.5	21.0	3.9	8.7	26.3
Mali	2015	97.9	98.8	65.4	76.1	2.7	8.8	23.0	6.9	3.2	10.8

Country	Year	Any breastfeeding (%)		Plain water (%)		Other liquids (%)		Other milks (%)		Complementary foods (%)	
		Milk-based only prelacteals	Water-based only prelacteals	Milk-based only prelacteals	Water-based only prelacteals	Milk-based only prelacteals	Water-based only prelacteals	Milk-based only prelacteals	Water-based only prelacteals	Milk-based only prelacteals	Water-based only prelacteals
Mauritania	2015	99.5	94.3	41.7	48.0	15.5	24.8	45.8	35.3	19.1	20.7
Mexico	2015	81.2	100.0	34.5	63.3	30.1	40.1	67.8	23.7	17.6	37.2
Moldova	2012	62.3	100.0	47.3	32.4	29.9	28.2	55.7	11.1	16.4	0.0
Mongolia	2018	97.9	100.0	28.0	34.4	10.6	11.0	47.0	36.2	26.9	19.7
Montenegro	2018	80.7	*	61.3	*	21.1	*	37.9	*	13.3	*
Mozambique	2011	94.3	100.0	25.2	67.6	22.1	11.5	47.5	6.4	17.3	34.1
Myanmar	2015	95.9	99.4	37.3	80.9	2.1	0.6	26.0	7.7	14.1	68.3
Namibia	2013	100.0	100.0	46.1	57.0	23.8	19.7	48.8	8.7	11.4	22.9
Nepal	2019	95.8	100.0	38.5	14.3	7.5	14.3	21.0	53.9	14.1	14.3
Niger	2012	98.1	98.3	73.1	91.5	15.2	11.0	10.0	3.5	10.9	8.9
Nigeria	2016	94.6	96.8	71.4	75.7	25.4	20.3	18.9	11.1	42.7	46.6
North Macedonia	2018	77.0	0.0	54.0	100.0	61.1	100.0	55.3	100.0	40.0	0.0
Pakistan	2012	95.5	95.8	37.3	31.2	3.2	4.0	45.7	34.6	8.9	12.7
Panama	2013	80.6	96.2	72.8	88.3	27.2	12.7	85.0	92.0	12.8	0.0
Paraguay	2016	84.8	92.6	51.9	32.4	11.9	27.7	59.2	26.5	8.8	23.8
Peru	2019	96.0	100.0	11.3	0.0	5.8	0.0	37.8	3.4	7.1	0.0
Sao Tome and Principe	2019	100.0	100.0	14.7	25.4	14.7	19.2	14.7	21.5	14.7	20.4
Senegal	2019	100.0	100.0	30.9	62.9	13.3	6.6	26.0	3.9	14.4	7.1
Serbia	2019	60.4	60.9	83.1	100.0	62.7	39.1	52.1	39.1	20.8	0.0
Sierra Leone	2017	100.0	96.4	0.0	63.8	0.0	3.9	39.2	18.9	0.0	18.6
State of Palestine	2019	89.2	95.6	26.3	20.0	27.5	24.4	49.5	36.7	24.3	21.2
Sudan	2014	98.1	97.1	45.4	45.5	6.7	10.5	26.2	10.0	8.2	12.5
Suriname	2018	74.6	100.0	88.7	100.0	17.5	0.0	71.2	53.7	48.3	37.3
Tajikistan	2012	100.0	96.7	57.6	77.9	0.0	6.8	56.7	22.3	14.3	8.3
Tanzania	2010	100.0	100.0	42.3	50.2	13.7	10.7	42.9	12.1	33.1	38.3
Thailand	2019	61.9	77.5	70.3	98.1	18.4	39.1	65.3	66.2	29.7	44.2

Country	Year	Any breastfeeding (%)		Plain water (%)		Other liquids (%)		Other milks (%)		Complementary foods (%)	
		Milk-based only prelacteals	Water-based only prelacteals	Milk-based only prelacteals	Water-based only prelacteals	Milk-based only prelacteals	Water-based only prelacteals	Milk-based only prelacteals	Water-based only prelacteals	Milk-based only prelacteals	Water-based only prelacteals
Togo	2017	100.0	98.6	36.0	40.3	0.0	0.0	33.9	1.6	15.5	3.5
Tonga	2019	79.4	100.0	15.5	36.1	5.1	0.0	84.1	0.0	12.8	0.0
Tunisia	2018	84.1	75.9	64.6	73.7	28.3	28.2	63.4	62.6	31.9	34.5
Turkey	2013	89.5	96.1	51.8	54.7	7.7	9.7	61.4	31.0	8.9	21.0
Ukraine	2012	63.5	95.6	83.6	100.0	24.3	45.8	68.5	51.0	24.3	59.9
Vietnam	2013	95.7	100.0	53.6	95.8	7.4	23.9	45.0	38.3	23.1	22.2
Yemen	2013	97.1	94.6	81.4	82.1	4.4	11.9	69.7	41.6	22.5	29.5
Zambia	2013	90.3	100.0	37.1	21.7	25.5	20.9	13.5	1.2	22.2	21.4
Zimbabwe	2019	100.0	94.1	72.9	83.6	10.5	27.6	19.2	0.0	36.0	49.9

[*]Due to the small number of children (<3) it was not possible to run the analysis. [**]Food groups: other liquids (sugar water, juices, liquid soups/ clear broth, and other liquids), other milks (formula or animal milks [cow, goat, etc.]), and complementary foods (baby food, flesh, eggs, vegetables, fruits, yogurt and dairy, tubers and grains, and other solid-semisolid foods).

Figure S1. Flow-chart of the selection of country surveys.

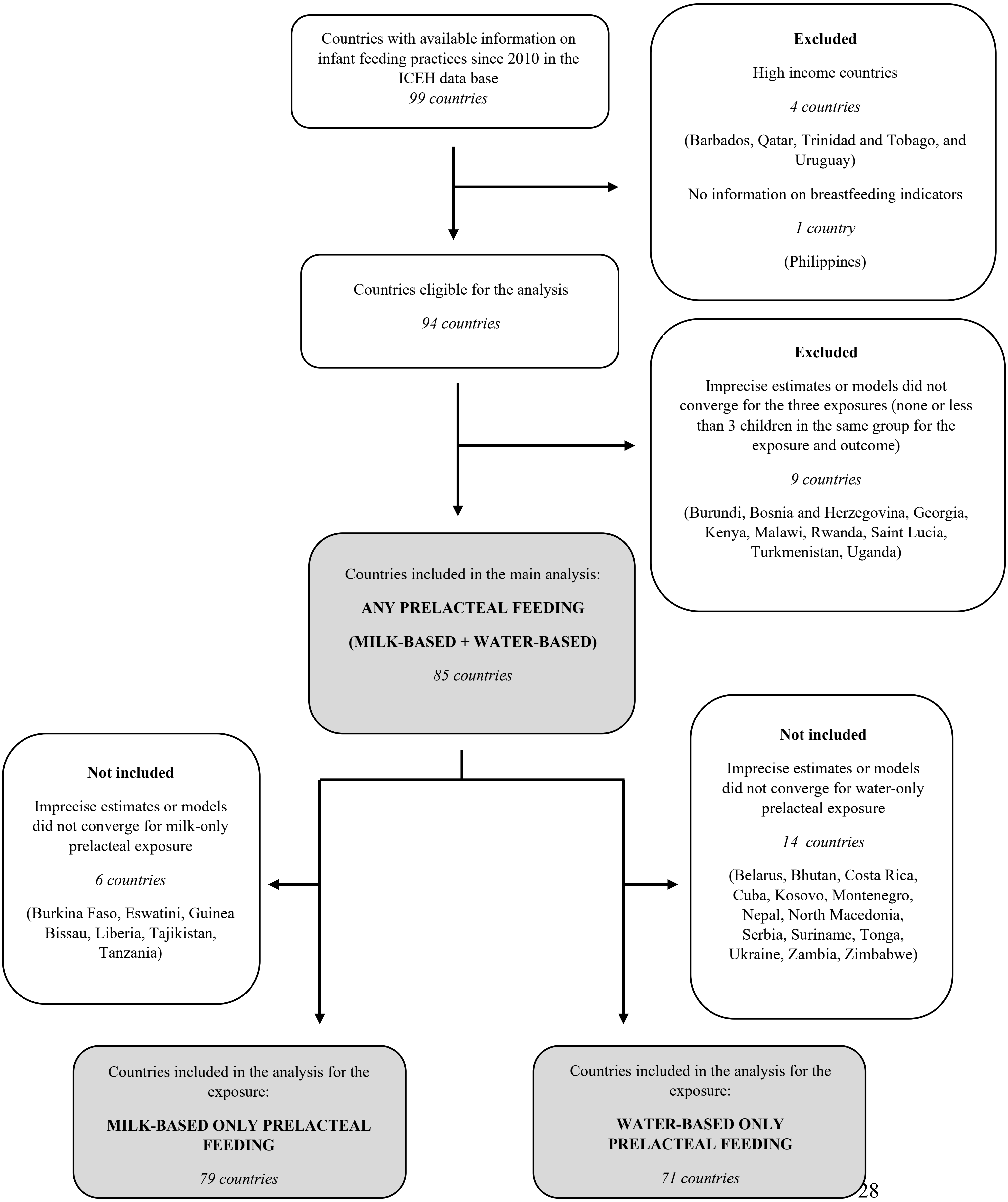

Figure S2. Proposed directed acyclic graph of the relationship between prelacteal feedings with exclusive breastfeeding and formula consumption under six months of age.

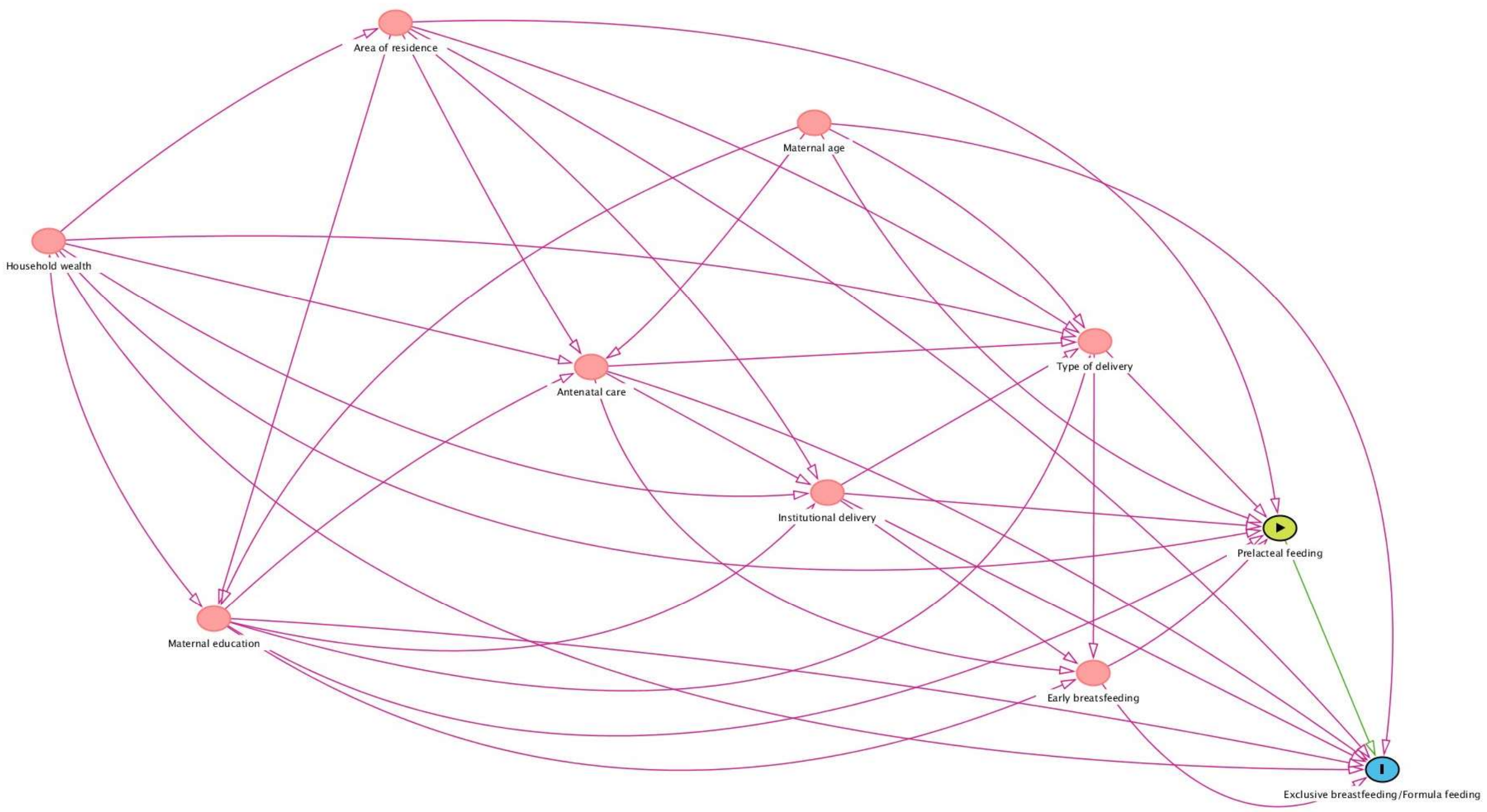

Figure S3. World map showing the countries included in the analysis.

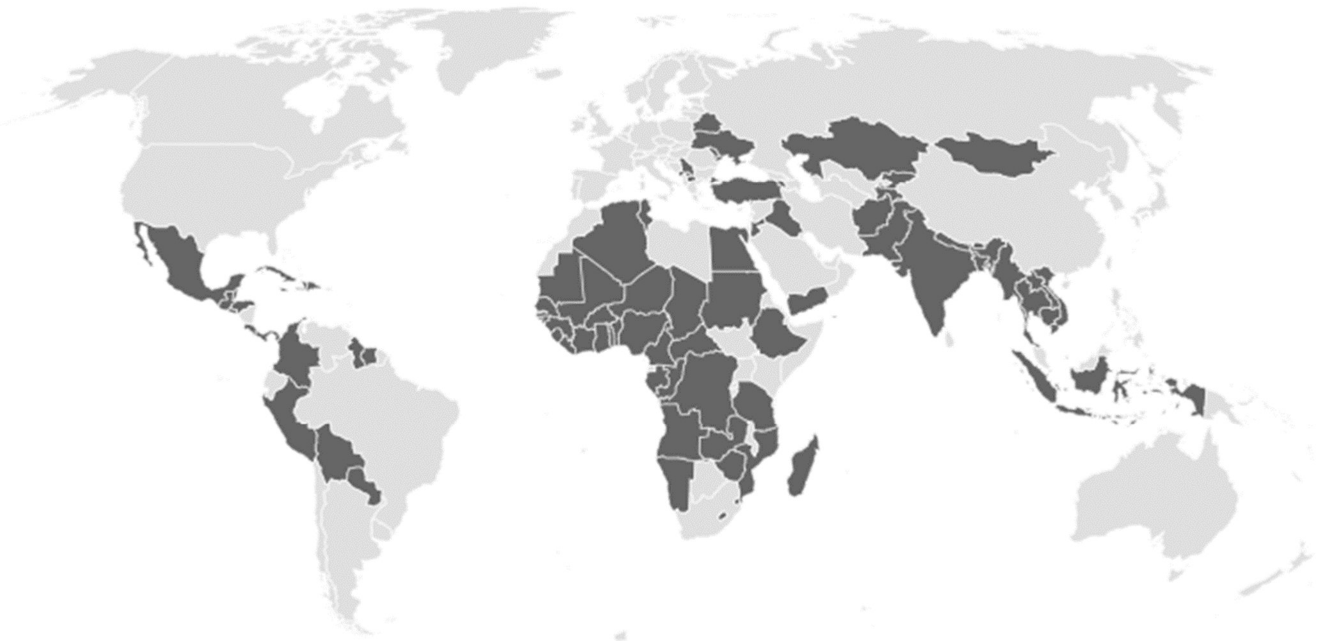

Figure S4. Pooled and country-specific prevalence ratios of the effect of milk-based only prelacteal feeding on exclusive breastfeeding.

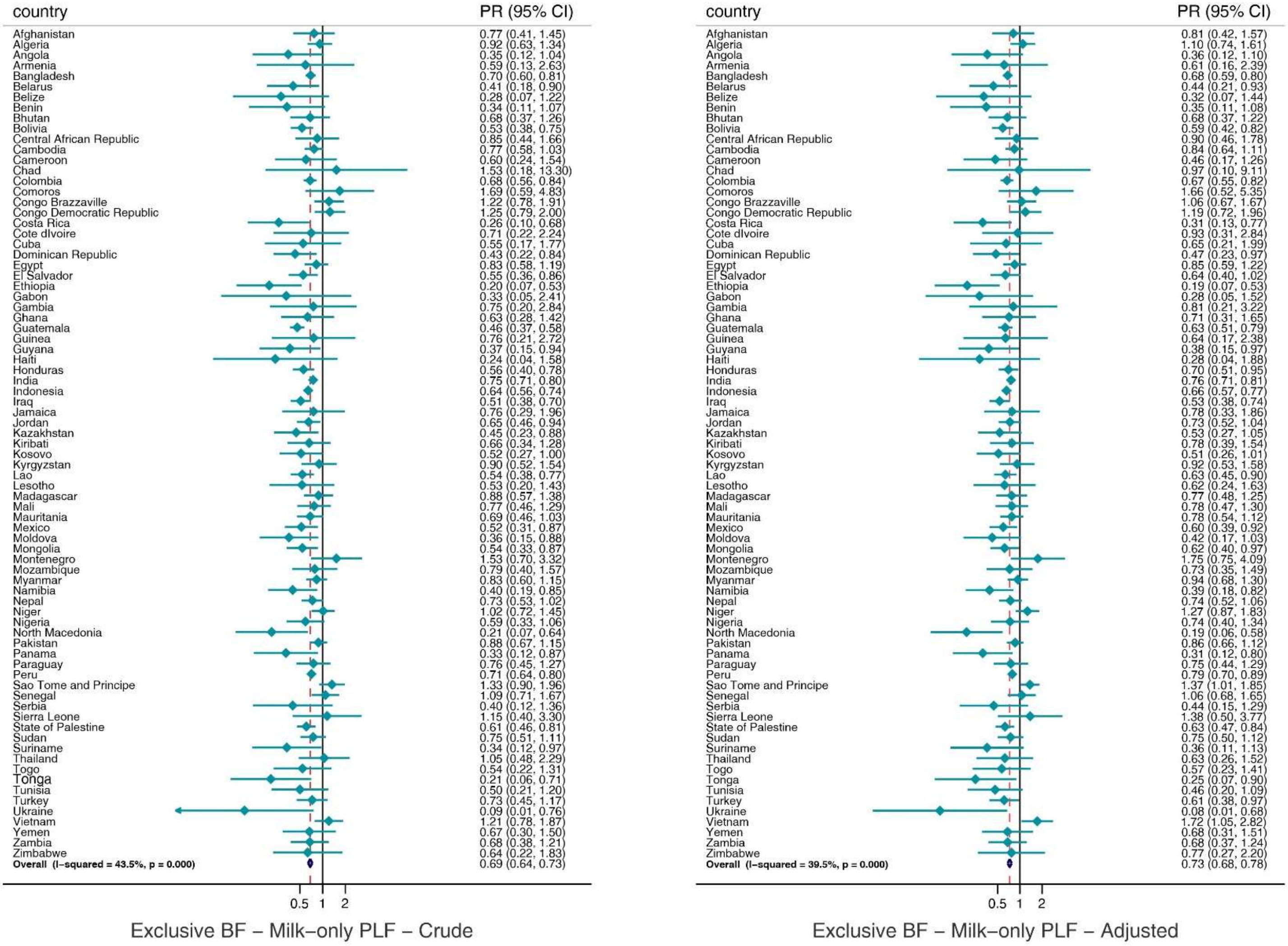

Legend: Exclusive BF - exclusive breastfeeding; Milk-only PLF – milk-based only prelacteal feeding.

Figure S5. Pooled and country-specific prevalence ratios of the effect of water-based only prelacteal feeding on exclusive breastfeeding.

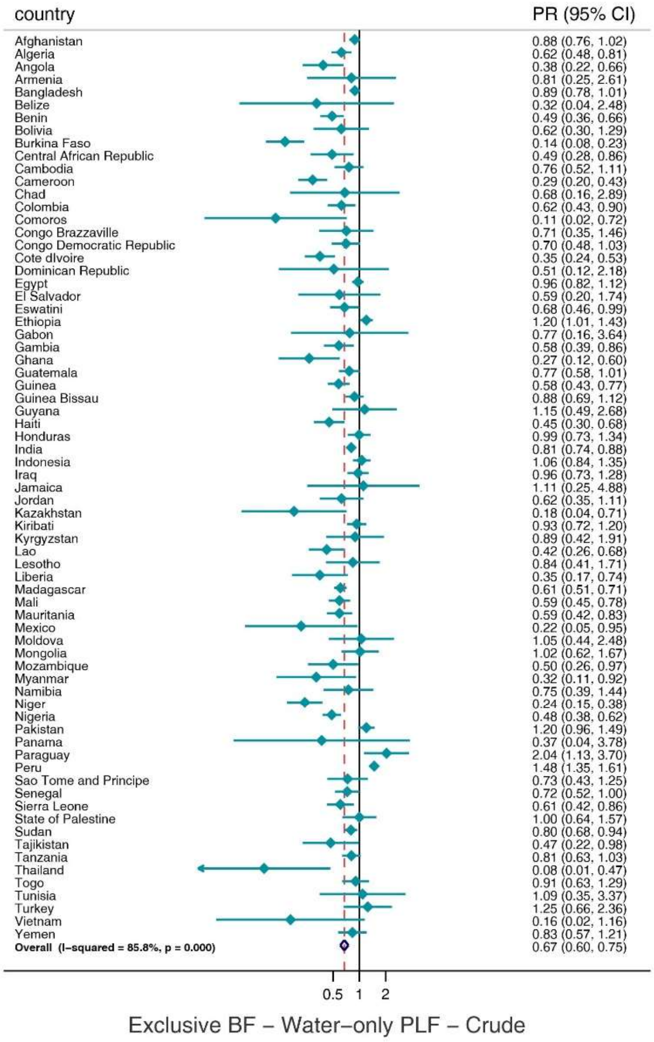

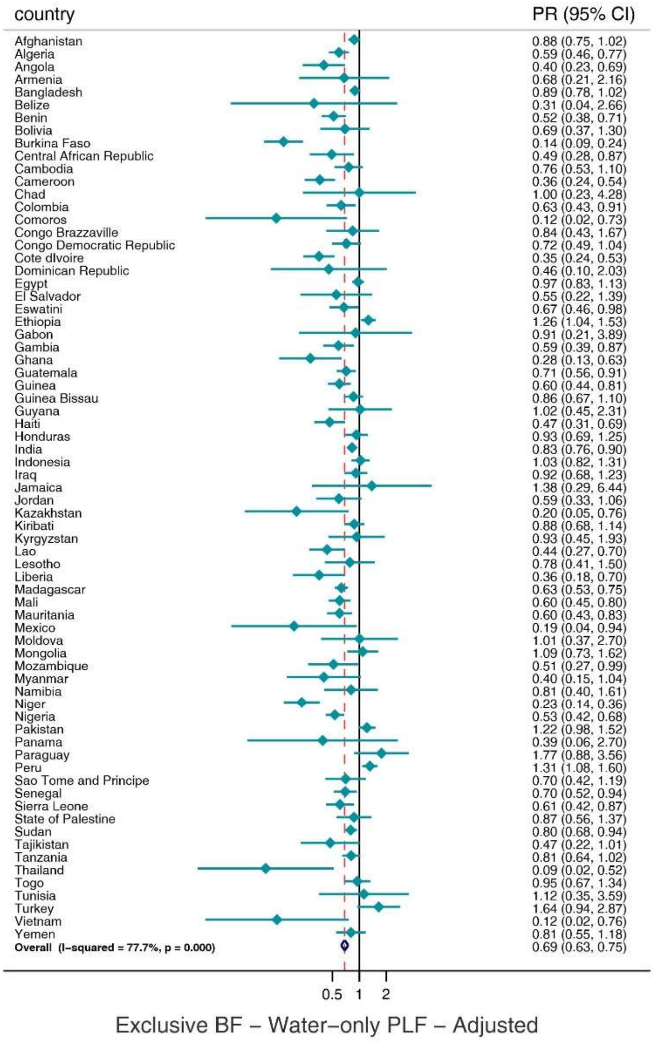

Legend: Exclusive BF - exclusive breastfeeding; Water-only PLF – water-based only prelacteal feeding.

Figure S6. Pooled and country-specific prevalence ratios of the effect of milk-based only prelacteal feeding on formula consumption.

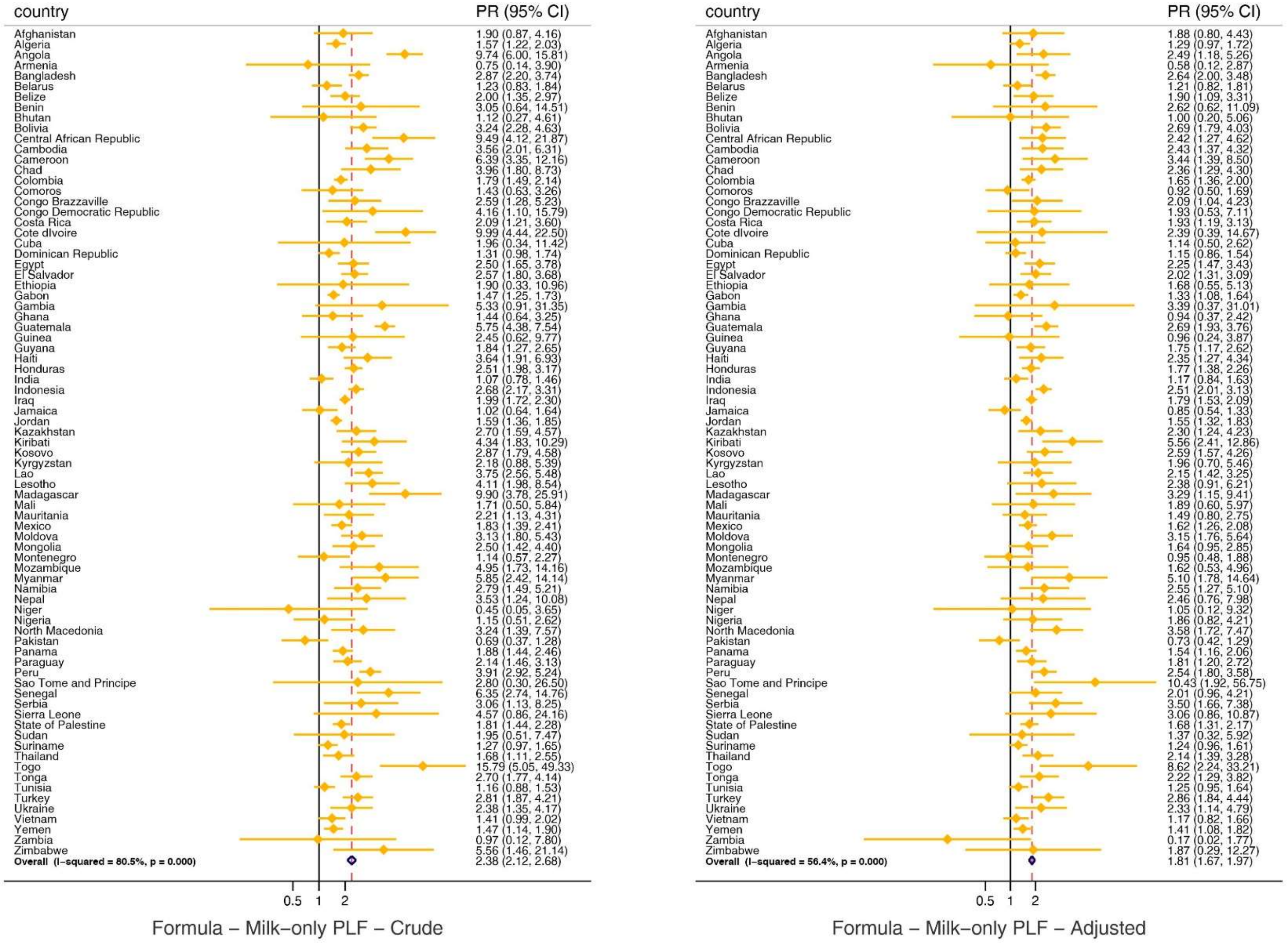

Legend: Formula – formula consumption; Milk-only PLF – milk-based only prelacteal feeding.

Figure S7. Pooled and country-specific prevalence ratios of the effect of water-based only prelacteal feeding on formula consumption.

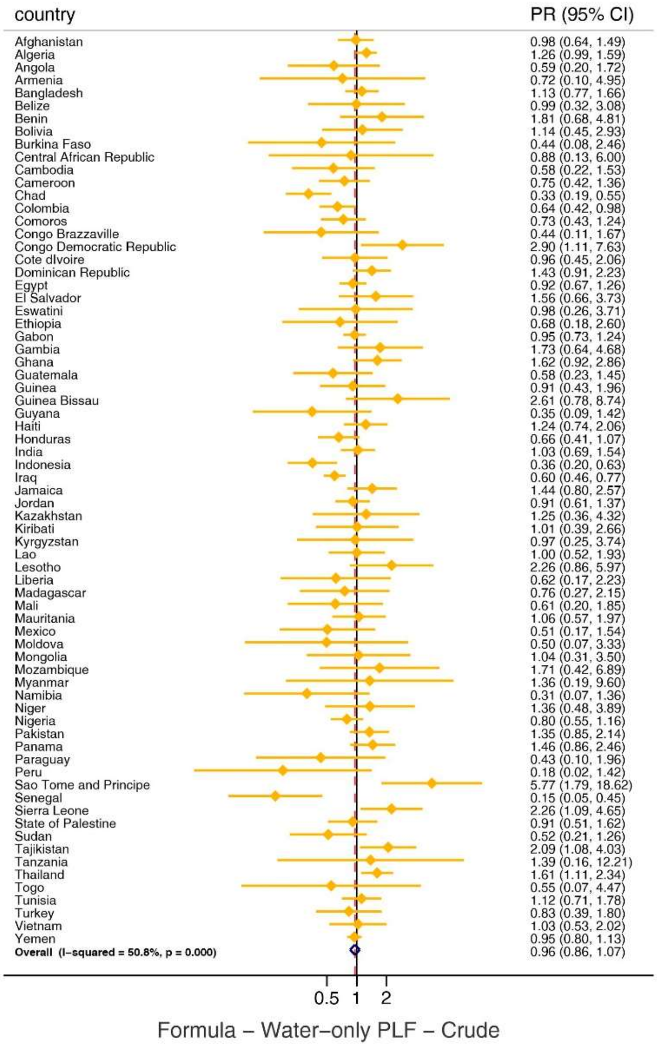

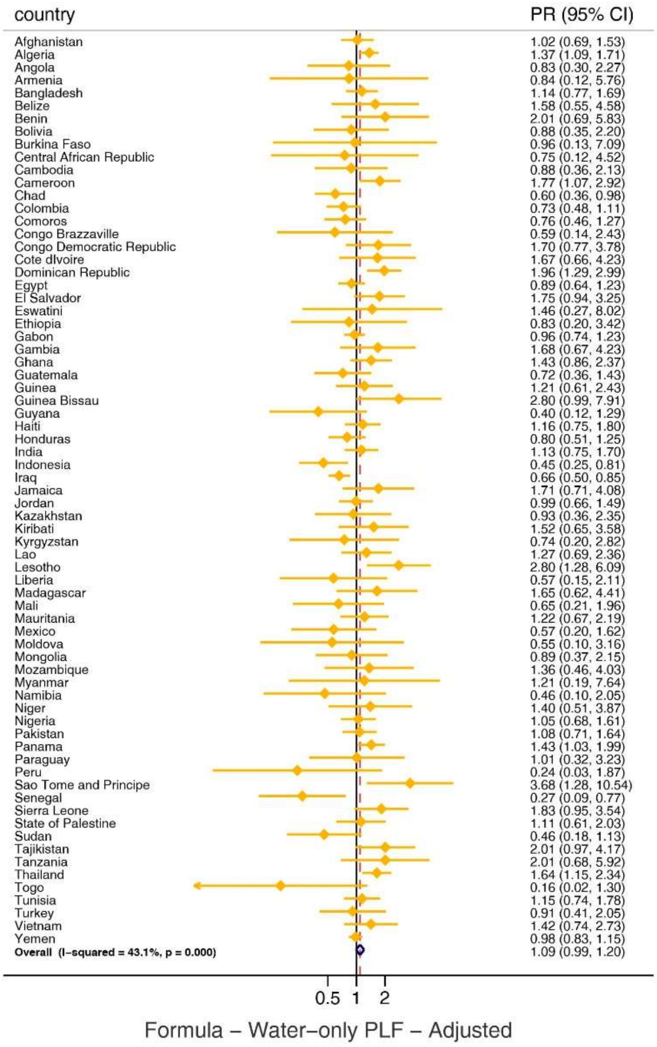

Legend: Formula – formula consumption; Water-only PLF – water-based only prelacteal feeding.
